# Fluorinated Phosphoadenosine 5′-Phosphosulfate Analogues for Continuous Sulfotransferase Activity Monitoring and Inhibitor Screening by ^19^F NMR Spectroscopy

**DOI:** 10.1021/acschembio.1c00978

**Published:** 2022-02-23

**Authors:** Agnieszka Mlynarska-Cieslak, Mikolaj Chrominski, Tomasz Spiewla, Marek R. Baranowski, Marcelina Bednarczyk, Jacek Jemielity, Joanna Kowalska

**Affiliations:** † Division of Biophysics, Institute of Experimental Physics, Faculty of Physics, University of Warsaw, Pasteura 5, 02-093 Warsaw, Poland; ‡ Centre of New Technologies University of Warsaw, Banacha 2c, 02-097 Warsaw, Poland

## Abstract

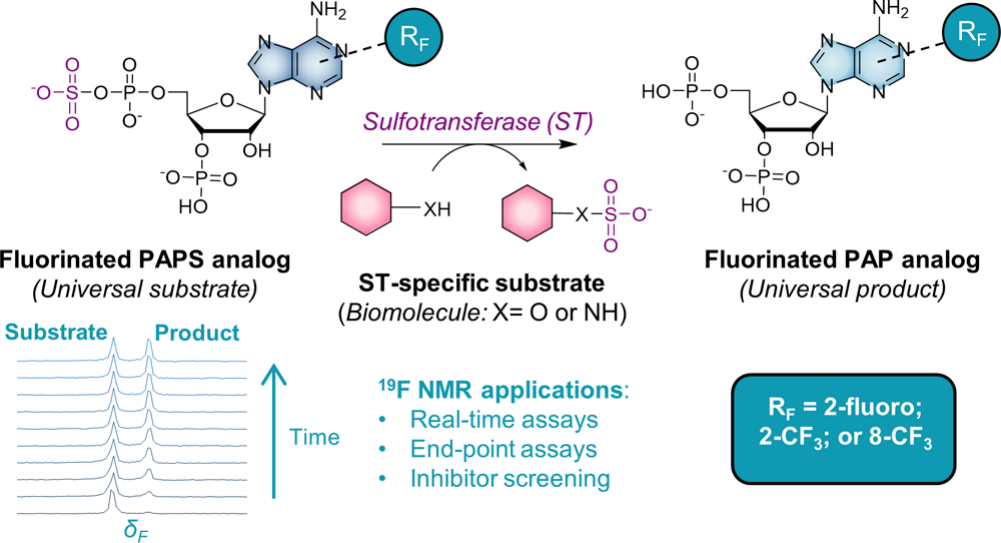

Sulfotransferases (STs) are ubiquitous enzymes that participate in a vast number of biological processes involving sulfuryl group (SO_3_) transfer. 3′-phosphoadenosine 5′-phosphosulfate (PAPS) is the universal ST cofactor, serving as the “active sulfate” source in cells. Herein, we report the synthesis of three fluorinated PAPS analogues that bear fluorine or trifluoromethyl substituents at the C2 or C8 positions of adenine and their evaluation as substitute cofactors that enable ST activity to be quantified and real-time-monitored by fluorine-19 nuclear magnetic resonance (^19^F NMR) spectroscopy. Using plant AtSOT18 and human SULT1A3 as two model enzymes, we reveal that the fluorinated PAPS analogues show complementary properties with regard to recognition by enzymes and the working ^19^F NMR pH range and are attractive versatile tools for studying STs. Finally, we developed an ^19^F NMR assay for screening potential inhibitors against SULT1A3, thereby highlighting the possible use of fluorinated PAPS analogues for the discovery of drugs for ST-related diseases.

## Introduction

**Figure 1:**
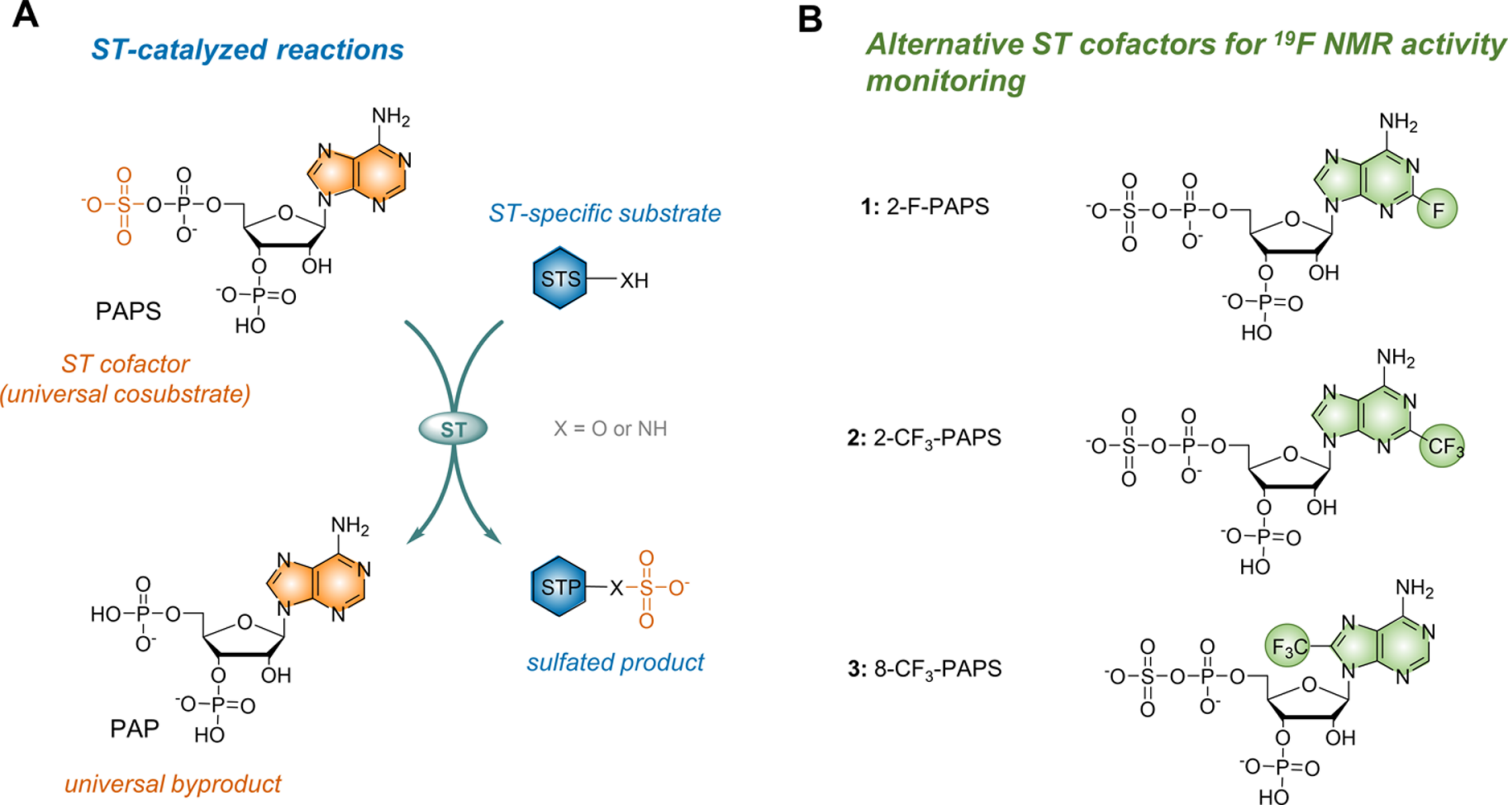
General reaction catalyzed by a PAPS-dependent sulfotransferase (ST) (A) and fluorinated PAPS analogues synthesized and evaluated in this study (B).

Sulfotransferases (STs) are enzymes that catalyze the transfer of sulfuryl (SO_3_) groups to various nucleophilic acceptors.^1^ Sulfotransferase-catalyzed reactions occur in all living domains, including bacteria, plants, and animals, and are involved in a variety of processes, such as enzyme regulation, detoxification, regulating hormonal balance, molecular recognition, and cellular signaling. Several human STs have been identified as biomarkers linked to cancer,^2^ neurodegenerative diseases,^3,4^ immune response effectiveness,^5^ and multiple other disorders.^6^ The vast majority of STs, including mammalian sulfotransferases (SULTs) and plant sulfotransferases (SOTs), use 3′-phosphoadenosine 5′-phosphosulfate (PAPS) as a universal cofactor, that is, a sulfate group donor. STs transfer the sulfuryl group from PAPS to acceptor molecules bearing O- or *N*-nucleophilic functional groups and release PAP as the byproduct (Figure 1A). ST substrate specificity varies from small molecules to complex macromolecules, including proteins and proteoglycans.

The biological importance of sulfotransferase-catalyzed reactions necessitates the development of robust assays for monitoring and screening sulfotransferase activity. Despite a number of methods developed with this goal in mind,^7^ the majority relies on the detection and quantification of enzyme-specific substrates,^8^ which significantly narrows their application scope.^9^ To the best of our knowledge, the only universal method for assaying STs involves the use of PAPS labeled with radioactive ^35^S [(^35^S)PAPS].^10^ Although the method is highly sensitive, the use of radioactive isotopes is limiting because it necessitates separating radioactive substrates and products and cannot be used to continuously monitor ST activity.

Fluorinated analogues of substrates for nucleotide-dependent enzymes have recently emerged as invaluable tools for enzymatic activity monitoring and inhibitor discovery by fluorine-19 nuclear magnetic resonance (^19^F NMR).^11−13^ For instance, 2-fluoro-ATP was shown to acts as a kinase substrate suitable for activity-based screening,^12^ whereas trifluoromethylated purine nucleotides were used to monitor activity of different phosphohydrolases.^13^ In this study, we aimed to develop a generally applicable ^19^F NMR-based assay employing fluorinated PAPS analogues suitable for sulfotransferase activity monitoring and screening both at a single time point and in real time. We envisaged that a fluorinated PAPS analogue that serves as an ST cosubstrate and is characterized by a ^19^F chemical shift different from that of the corresponding PAP analogue can be used to monitor reaction progress by ^19^F NMR spectroscopy. To explore this idea, we synthesized three PAPS analogues bearing fluorine or trifluoromethyl substituents at the C2- or C8-positions of the adenine moiety (Figure 1B). We characterized the ^19^F NMR-related properties of the compounds and evaluated them as cosubstrates for two model sulfotransferases, namely the plant sulfotransferase AtSOT18 (involved in plant signaling) and mammalian sulfotransferase SULT1A3 (involved in the metabolism of biogenic amines).^14,15^

We found that the compounds have promising substrate- and ^19^F-NMR-related properties and thereby are potentially versatile molecular tools for STs. One of the compounds was successfully used to screen a library of 59 ligands against SULT1A3.

## Results and Discussion

**Scheme 1:**
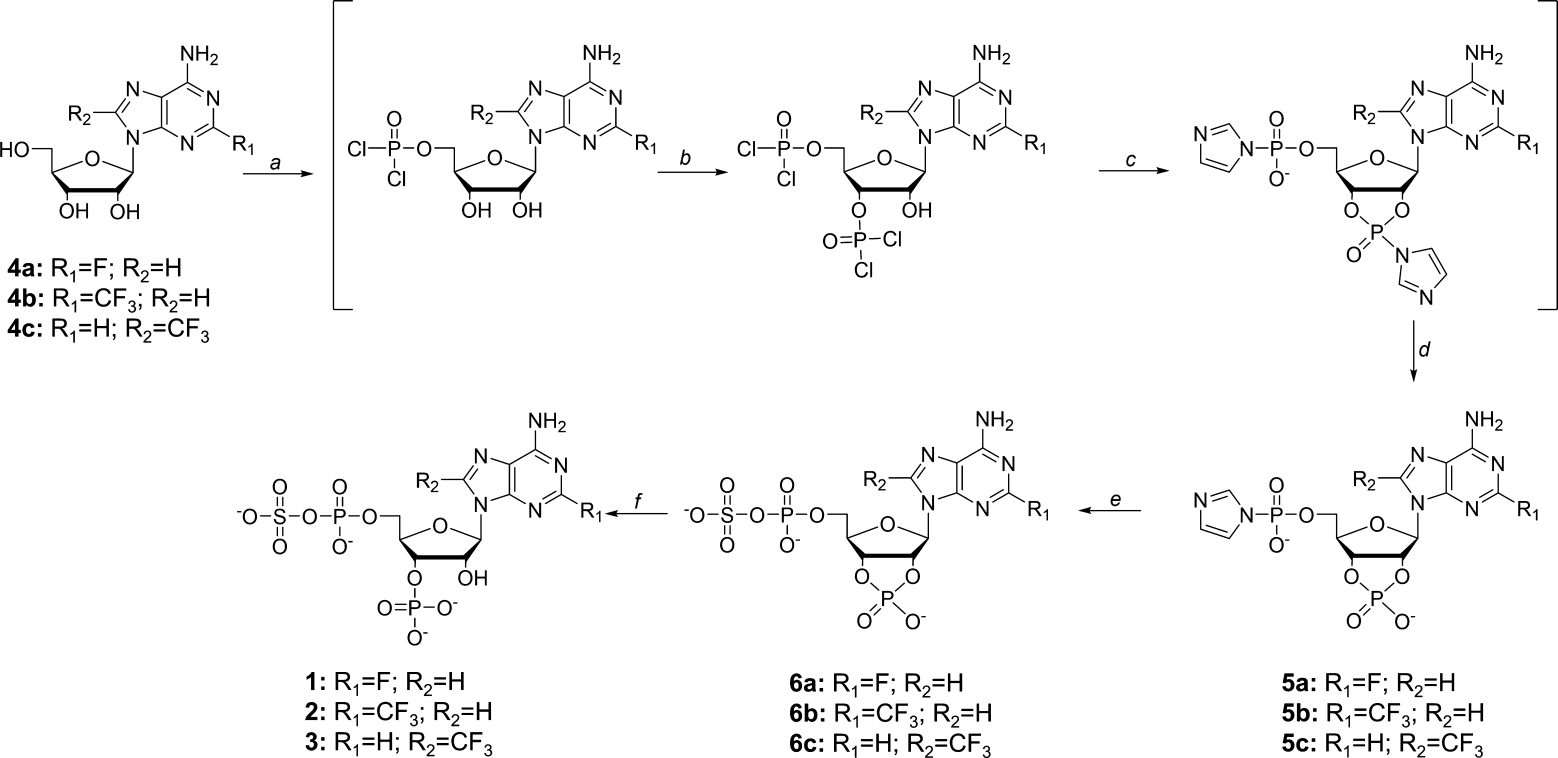
Synthesis of Fluorinated PAPS Analogues **1–3** Reaction conditions: (a) POCl_3_, PO(OCH_3_)_3_ at 0 or −5 °C and 2,6-lutidine (for **4b**, **4c**). (b) 1. POCl_3_; 2. precipitation with Et_2_O. (c) Imidazole, PO(OCH_3_)_3_. (d) H_2_O. (e) Bis(tributylammonium) sulfate, MgCl_2_, DMF. (f) RNAse T2 (50 mM ammonium acetate buffer, pH 7.3, 37 °C, 300 rpm), 24 h.

To investigate the development of a ^19^F-labeled cofactor for sulfotransferases, we designed three fluorinated PAPS analogues as potential sulfotransferase substrates (Figure 1B; compounds **1–3**). Analogue **1** has a single fluorine substituent at the C2-position of adenine, whereas **2** and **3** each contains a trifluoromethyl group at either the C2- or C8-position. We aimed to compare the properties of the monofluoro- and trifluoromethyl-substituted compounds, as the presence of three equivalent fluorine atoms should increase the sensitivity and quality of the ^19^F NMR signal; however, it may affect the shape and conformation of the PAPS molecule to some extent, possibly resulting in a broader range of ST enzymes rejecting the modified compound as a cofactor. We also attempted to synthesize 8-fluoro-substituted PAPS but found that 8-fluoroadenosine is highly susceptible to depurination, which made the synthesis impossible to complete. Compounds **1–3** were synthesized in several chemical steps including a final enzymatic cleavage step (Scheme 1). Unprotected fluorinated nucleosides **4a–c** used as starting materials were either obtained from commercial sources (**4a**) or synthesized by recently reported procedures (**4b, 4c**).^13,16^

The 5′-phosphorimidazolides of appropriate 2′,3′-cyclophosphonucleosides were crucial synthetic intermediates and were prepared using a one-pot three-step procedure (**5a–c**; Scheme 1). To that end, a suspension of **4a–c** (1 equiv) in trimethyl phosphate was treated with anhydrous POCl_3_ (3 equiv) at −5 or 0 °C. The addition of 3 equiv of 2,6-lutidine was required for **4b** and **4c** to avoid depurination. The reaction was allowed to proceed until reversed-phase high-performance liquid chromatography (RP-HPLC) revealed complete conversion of the substrate to the corresponding 5′-phosphorylated product (approximately 3–4 h). A second portion of POCl_3_ (6 equiv) was then added, and the reaction was allowed to proceed (usually overnight) to enable phosphorylation at the 2′/3′-position of the nucleoside (see the Supporting Information for details). The diphosphorylated intermediate was precipitated with cold diethyl ether and centrifuged, after which it was re-dissolved in trimethyl phosphate (to a concentration of ∼100 mM), excess imidazole was added, incubated for 1 h at RT, and then hydrolyzed. The resulting products **5a–c** were purified by ion-exchange chromatography and stored as DMF solutions to avoid decomposition, which occurred when we attempted to isolate them in the solid form. Compounds **5a–c** were then coupled with tributylammonium sulfate (4 equiv) in DMF in the presence of excess MgCl_2_ (8 equiv).^17^ These reactions were complete within 18 h with conversions of 27–52%, as determined by RP-HPLC. The product was purified by ion-exchange chromatography (DEAE Sephadex) followed by semi-preparative RP-HPLC. Finally, the 2′,3′-cyclophosphate was enzymatically cleaved to the 3′-phosphate in high yield (∼90%) using RNAse T2.^18^

**Figure 2:**
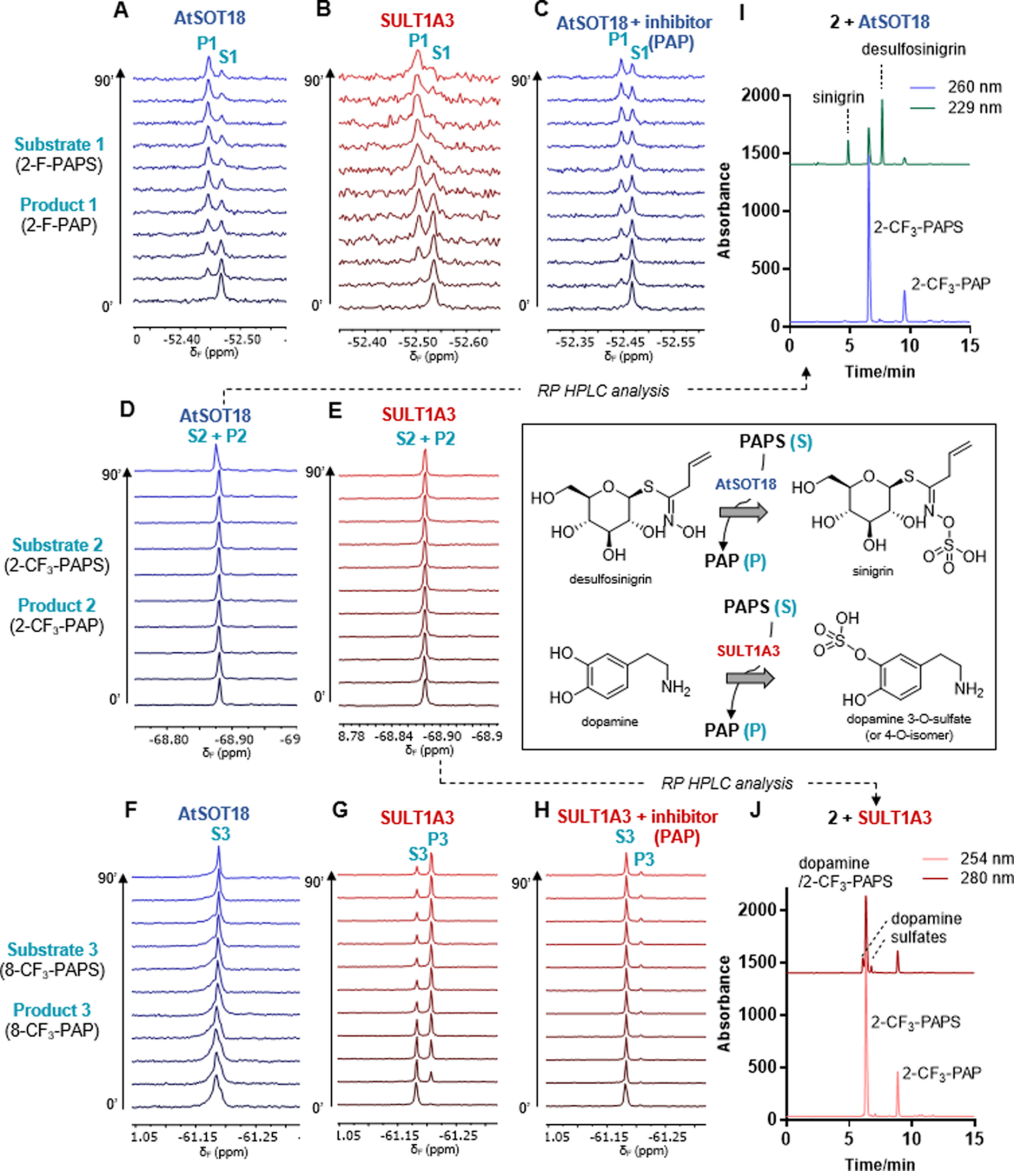
Evaluating fluorinated PAPS analogues as universal sulfotransferase substrates by ^19^F NMR spectroscopy. (A) 2-F-PAPS with AtSOT18; (B) 2-F-PAPS with SULT1A3; (C) 2-F-PAPS with AtSOT18 and a 200 μM inhibitor (PAP); (D) 2-CF_3_-PAPS with ATSOT18; (E) 2-CF_3_-PAPS with SULT1A3; (F) 8-CF_3_-PAPS with AtSOT18; (G) 8-CF_3_-PAPS with SULT1A3; (H) 8-CF_3_-PAPS with SULT1A3 and a 200 μM inhibitor (PAP); (I) RP-HPLC trace of the AtSOT18-catalyzed reaction in the presence of 2-CF_3_-PAPS after 60 min; and (J) RP-HPLC trace of the SULT1A3-catalyzed reaction in the presence of 2-CF_3_-PAPS after 60 min. The inset shows reactions catalyzed by both enzymes. Reaction conditions: AtSOT18 −200 μM PAPS analogue, 200 μM desulfosinigrin, 100 nM enzyme in 83 mM Tris, pH 9.0, containing 9.2 mM MgCl_2_, and 10% D_2_O at 37 °C; SULT1A3 −200 μM PAPS analogue, 200 μM dopamine, 100 nM enzyme in 6.7 mM K_2_HPO_4_, pH 7.4, containing 10% D_2_O at 37 °C.

We next examined how well PAPS analogues **1–3** were accepted as cofactors in sulfotransferase-catalyzed biochemical reactions. To this end, each compound **1–3** was incubated with model sulfotransferases and their specific substrates under conditions previously found to be optimal for these enzymes, with reaction progress monitored independently by ^19^F NMR spectroscopy (Figure 2A–H) and RP-HPLC (Figures 2I,J; S1, S2). As model enzymes, we chose two sulfotransferases of different origin and substrate specificity: plant AtSOT18^14,19^ and human SULT1A3.^20^ AtSOT18 participates in the biosynthesis of glucosinolates that are precursors of mustard oils and are responsible for the properties of many pungent plants,^21^ while SULT1A3 is a human enzyme involved in the metabolism of endogenous catecholamines (e.g., dopamine and epinephrine), serotonin, and structurally related xenobiotics.^22^ SULT1A3 has been shown to protect neurons from dopamine cytotoxicity and has been linked to neurodegenerative diseases and liver cancer.^4,23^

A reaction mixture containing 2-F-PAPS (**1**), either AtSOT18 or SULT1A3, and the corresponding ST-specific substrate (desulfosinigrin or dopamine, respectively) was monitored by ^19^F NMR spectroscopy, which revealed that **1** is accepted by each enzyme as a cofactor. Each ^19^F NMR spectrum consisted of a single signal at approximately −52.5 ppm that corresponds to 2-F-PAPS prior to the addition of the enzyme (Figure 2; the chemical shifts are slightly different for the two enzymes due to different buffer compositions). The signal corresponding to 2-F-PAPS gradually decreased in the presence of the enzyme, while a new signal that was downfield shifted by 0.02 ppm emerged (Figure 2A,B). This new signal increased slowly in the presence of PAP, the natural sulfotransferase inhibitor (Figure 2C), and was not observed in the absence of the enzyme (Figure S3), suggesting that it corresponds to 2-F-PAP, the enzymatic reaction byproduct. RP-HPLC supported by electrospray ionization mass spectrometry confirmed that 2-F-PAPS had been converted into 2-F-PAP and that sulfotransferase-specific products had formed: sinigrin for AtSOT18 (Figure S1) or two isomers of dopamine sulfate for SULT1A3 (Figure S2). In addition, these experiments also revealed that 2-F-PAPS has substrate properties comparable to those of unmodified PAPS.

In contrast, the ^19^F NMR spectrum of 2-CF_3_-PAPS (**2**) incubated with either AtSOT18 or SULT1A3 did not change over time (Figure 2D,E), which may indicate that either 2-CF_3_-PAPS is not accepted as a cosubstrate by the enzyme or that 2-CF_3_-PAPS and 2-CF_3_-PAP signals overlap under the experimental conditions. RP-HPLC of analogously prepared samples incubated for 1 h with each enzyme revealed the formation of the corresponding sulfated product, albeit with lower efficiency than that of PAPS (Figures 2I,J; S1D, S2D). This clearly indicates that while compound **2** is a substrate for AtSOT18 and SULT1A3, the reaction progress cannot be continuously monitored by ^19^F NMR spectroscopy under the applied conditions.

Interestingly, 8-CF_3_-PAPS (**3**) was recognized differently by the above-mentioned two enzymes. The formation of the 8-CF_3_-PAP analogue was not observed by ^19^F NMR spectroscopy in the presence of AtSOT18, whereas two signals with intensities that changed over time were clearly visible in the presence of SULT1A3 (Figure 2F–H). RP-HPLC of the reaction mixture after incubating with AtSOT18 or SULT1A3 for 1 h confirmed that 8-CF_3_-PAPS is a good substrate for SULT1A3 (comparable to PAPS) under the studied conditions but not for AtSOT18 (Figures S1, S2).

Overall, these studies revealed that all three compounds are potential molecular tools for sulfotransferase research; however, each is associated with some limitations. 2-F-PAPS serves as a substrate for each enzyme, with the signal of the fluorinated substrate well resolved from that of the product (2-F-PAP) in each case. However, because only a single fluorine atom is used as the ^19^F label, the spectra acquired using 2-F-PAPS are of lower quality (signal-to-noise ratio) than those obtained using 2-CF_3_-PAPS or 8-CF_3_-PAPS at the same concentration. 2-CF_3_-PAPS is a substrate for both enzymes and produces spectra of good quality; consequently, it is a good potential universal sulfotransferase co-factor candidate. Unfortunately, the ^19^F signals of 2-CF_3_-PAPS and 2-CF_3_-PAP overlap under the studied conditions; hence, monitoring reaction progress by ^19^F NMR spectroscopy is impossible. Finally, 8-CF_3_-PAPS provided spectra of good quality with a signal that was well-resolved from that of 8-CF_3_-PAP, but the compound was only accepted as a substrate by SULT1A3 and not AtSOT18; hence, it may be of limited applicability compared to other two compounds.

**Figure 3:**
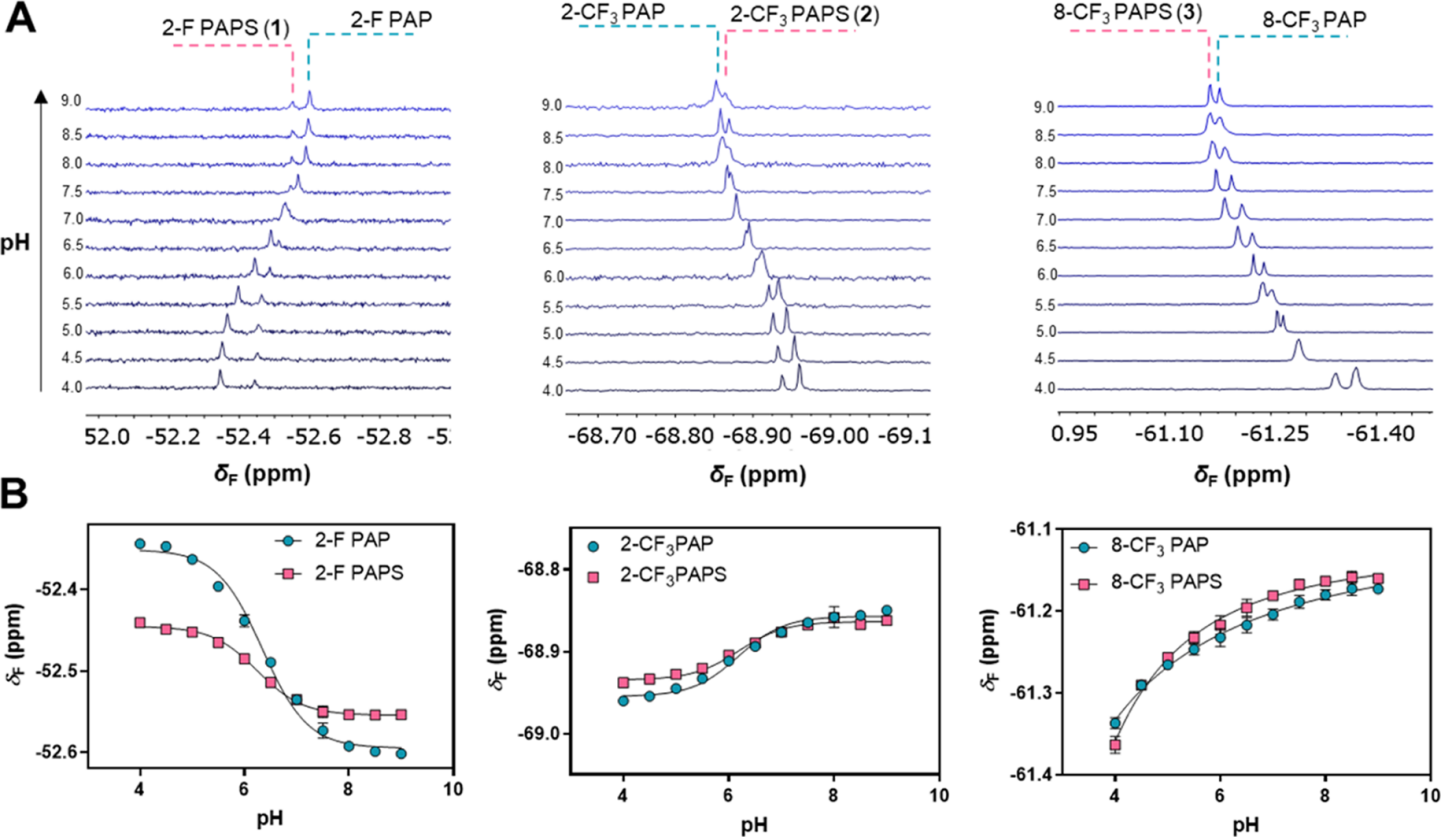
pH-dependent changes in δ_F_ for PAPS analogues **1–3** and the corresponding PAP analogues. (A) Representative ^19^F NMR [471 MHz, 200 μM total nucleotides in 6.7 mM K_2_HPO_4_ with 10% of D_2_O (v/v), 37 °C] spectra from titration experiments of PAPS/PAP analogue mixtures. (B) Titration curves obtained from duplicate experiments. Data points are means ± standard deviations. Solid lines are theoretical curves fitted to the data points (see the Experimental Section).

The ^19^F signal-separation problem observed for 2-CF_3_-PAPS and 2-CF_3_-PAP encouraged us to systematically search for conditions under which the resonances corresponding to the PAPS analogues and their corresponding PAP counterparts are well resolved. We hypothesized that the chemical shifts of the signals corresponding to the PAPS and PAP analogues may be affected by their protonation state. Because PAPS contains a single ionizable group at pH ≈ 7 (3′-phosphate) and PAP contains two such groups (3′-phosphate and 5′-phosphate; Figure S4), different pH-dependent effects may be observed for the two compounds. Consequently, we investigated chemical shifts as functions of solution pH (Figure 3). To that end, aqueous solutions of PAPS analogues **1–3** were subjected to acidic hydrolysis to obtain non-equimolar mixtures of PAPS and PAP analogues. The pH of each mixture was then adjusted to 4 and step-wise titrated with aqueous NaOH to pH 9 (in ∼0.5 pH unit steps). The ^19^F NMR spectrum of the mixture was recorded at each step, and the δ_F_ values of the PAPS and PAP analogues were plotted as functions of pH. Interestingly, each compound exhibited unique pH-dependent properties, with the 2-F-PAPS/2-F-PAP pair being most sensitive to pH change (Figure 3).

The signals for the 2-F-PAPS/2-F-PAP pair were well resolved at pH values in the 4.0–6.0 range (Δδ_F_ ppm: 0.05–0.1) and between 8.0 and 9.0 (Δδ_F_ 0.05 ppm), whereas separation was less efficient at pH 6.5–7.5, with almost complete overlap observed at pH 7.0. The 2-CF_3_-PAPS/2-CF_3_-PAP pair was the least sensitive to pH change and also provided the poorest signal separation. Nonetheless, sufficient signal separation was achieved at pH 4.0–5.5 (Δδ_F_ 0.01–0.02 ppm), with slightly poorer separation observed at pH 8.5–9.0 (Δδ_F_ ppm: 0.01). In contrast, the signals associated with the 8-CF_3_-PAPS/8-CF_3_-PAP pair were resolved similarly well over a wide pH range (6.0–9.0; Δδ_F_ ppm: 0.01–0.03). Considering that most sulfotransferases exhibit optimum activity under neutral or slightly alkaline conditions and that the phosphosulfate bonds in PAPS are susceptible to acidic hydrolysis, we recommend optimum pH ranges for these analogues (Table 1).

**Table 1:** Optimized Conditions for ^19^F NMR-Monitored Sulfotransferase Experiments^a^

		AtSOT18			
compound	recommended assay type and pH range^b^	assay type	buffer	δ_F_ PAPS analogue (ppm)	δ_F_ PAP analogue (ppm)
**1** (2-F-PAPS)	real-time, end-point(discontinuous),pH 7.5–9.0	real-time	83 mM tris pH 9.0, 9.2 mM MgCl_2_ with 10% D_2_O	–52.47	–52.45
**2** (2-CF_3_-PAPS)	end-point, 8.5–9.0	end-point	reaction: 83 mM tris, pH 8.5, 10% D_2_O readout: 1:1 mixture (v/v) of reaction buffer and acetonitrile	–68.76	–68.74
**3** (8-CF_3_-PAPS)	real-time, end-point, pH 6.5–9.0	real-time	not active^c^	–61.01	not active

		SULT1A3			
compound	recommended assay type and pH range^b^	assay type	buffer	δ_F_ PAPS analogue (ppm)	δ_F_ PAP analogue (ppm)
**1** (2-F-PAPS)	real-time, end-point(discontinuous),pH 7.5–9.0	real-time	6.7 mM K_2_HPO_4_, pH 6.5	–52.53	–52.50
**2** (2-CF_3_-PAPS)	end-point, 8.5–9.0	end-point	reaction: 6.7 mM K_2_HPO_4_, pH 7.4 readout: 1:1:1 mix of reaction buffer, ACN and 30 mM K_2_HPO_4_, pH 9.5, 10% D_2_O (final pH 8.5)	–68.77	–68.75
**3** (8-CF_3_-PAPS)	real-time, end-point, pH 6.5–9.0	real-time	6.7 mM K_2_HPO_4_, pH 7.4, 10% D_2_O	–61.18	–61.20

a Representative ^19^F NMR spectra acquired under these conditions are shown in Figures 4 and S5.

b Optimal pH for ^19^F NMR monitoring/readout.

c Compound **3** is not a substrate for AtSOT18.

The pH studies explain why good signal separation was not observed for enzymatic reactions conducted in the presence of 2-CF_3_-PAPS at pH 7.4 (reaction with SULT1A3) but do not account for the lack of separation observed at pH 9.0 (for AtSOT18). We speculate that the presence of protein and buffer components additionally contributes to ^19^F NMR signal broadening that hampers signal separation. Therefore, we investigated the development of a discontinuous (endpoint) assay, in which different conditions were used for the enzymatic reaction and ^19^F NMR measurements. After optimization studies, we found that adding 50% acetonitrile to the reaction mixture causes protein denaturation and in consequence improves the signal shape. This finding enabled the development of discontinuous assay conditions for 2-CF_3_-PAPS and both AtSOT18 and SULT1A3 enzymes, thereby establishing the final set of conditions recommended for all compounds and each enzyme (Figures 4, S5; Table 1). Therefore, despite 2-CF_3_-PAPS having limited applicability for real-time ST-activity monitoring, it may still be useful for various experiments, such as end-point analyses and activity-based screening assays.

**Figure 4:**
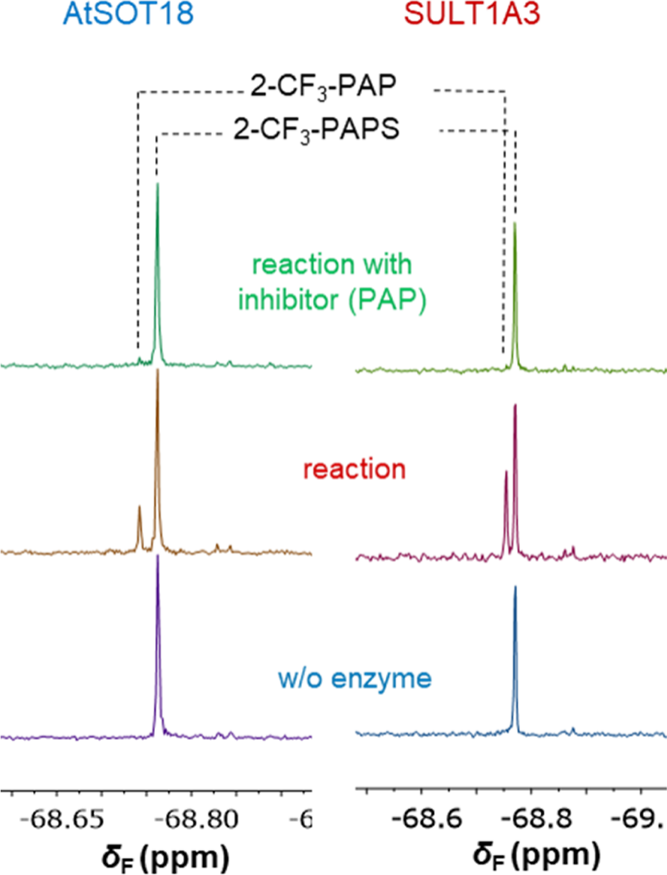
Endpoint ^19^F NMR assay overview. Spectra of ST-catalyzed reactions acquired using 2-CF_3_-PAPS under optimized conditions (showcased in Table 1) at the 40 min timepoint.

**Figure 5:**
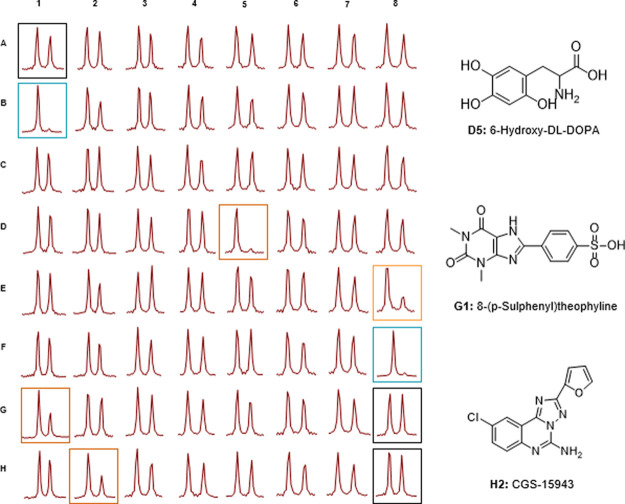
^19^F NMR-based screening of a small library of pharmaceutically active compounds against SULT1A3. Samples A1, G8, and H8 (black frames): positive controls (reactions without an inhibitor): samples B1 and F8 (blue frame): negative controls (reactions without an enzyme). Sample E8 (orange frame): inhibition control (reaction in the presence of PAP). Samples D5, G1, and H2: hits confirmed by duplicate screening (Table S1).

Finally, we performed a proof-of-concept SULT1A3 activity-screening experiment to demonstrate the practical utility of fluorinated PAPS analogues. To that end, we selected a subset of compounds from the LOPAC1280 library and added them to SULT1A3-catalyzed reactions in the 96-well format in the presence of 8-CF_3_-PAPS. Control reactions included samples without an inhibitor, without a protein, and with the addition of a natural ST inhibitor (PAP). All reactions were terminated at a single time point (40 min), transferred to NMR tubes, and subjected to ^19^F NMR spectroscopy to assess inhibition percentage (Figure 5; Table S1). Three hits were identified among the 59 screened compounds: 6-hydroxydopamine, which had been previously identified as a competitive inhibitor of SULT1A3/4,^24^ and 8-(*p*-sulfophenyl)teophiline and CGS-15943, which have not been previously studied in the context of STs but had been identified as adenosine receptor antagonists.^25^

## Conclusions

2-F-PAPS (**1**), 2-CF_3_-PAPS (**2**), and 8-CF_3_-PAPS (**3**) were investigated as PAPS substitutes in ST-catalyzed reactions. The compounds were synthesized from unprotected nucleosides in a straightforward manner that can provide access to sufficient quantities for large-scale applications. Substrate properties of the compounds were evaluated in the context of two unrelated enzymes (AtSOT18 and SULT1A3), which revealed that all three compounds are promising molecular tools for sulfotransferases. 2-F-PAPS (**1**) and 2-CF_3_-PAPS (**2**) were accepted as co-factors by both studied enzymes, which is a good premise for potential broad applicability, and enabled reaction progress to be monitored by ^19^F NMR spectroscopy either in a real-time or in discontinuous format. 8-CF_3_-PAPS (**3**) was well accepted as a substrate by SULT1A3 but not AtSOT18, which suggests that it may be compatible with a more limited range of enzymes.

Because the vast majority of sulfotransferases are PAPS-dependent enzymes, the ^19^F NMR assays reported herein are potentially generally applicable, being possibly limited only by the PAPS recognition requirements of the particular enzymes. Although screening against a broader panel of sulfotransferases and the determination of kinetic parameters should be performed in the future to explore the full potential and limitations of those novel tools, previous structural studies indicate that the PAPS-binding site is conserved between different STs,^1,26^ strongly suggesting broader applicability of our findings. Future studies should take into account that the *K*_M_ values of unmodified PAPS for different sulfotransferases vary from medium nanomolar to medium micromolar values, and if the *K*_M_ of the PAPS analogue is significantly lower than its concentration used in the assay, this will bias the sensitivity of the assay toward uncompetitive and non-competitive inhibitors.^27^

Overall, our study revealed that fluorinated PAPS analogues are promising molecular tools for studying sulfotransferase activity by ^19^F NMR spectroscopy. To the best of our knowledge, this study demonstrated the first proof of concept of a universal (i.e., PAPS-based) ST assay that does not rely on radioactivity. Because contemporary ^19^F NMR-based assays are highly versatile, the developed approach may benefit various ST-related research areas, including ST-specific substrate specificity profiling, comparing ST isoform activities, activity-based inhibitor screening against therapeutically relevant sulfotransferases, or assaying the ST activities in complex biological mixtures (e.g., cell lysates or tissue homogenates).

## Methods

### General Information

^19^F NMR spectra were recorded with a BRUKER AVANCE III HD spectrometer equipped with PA BBO 500S1 BBF-H-D-05 Z SP probe at 471 MHz (probe sensitivity: 550 S/N ratio for 0.05% trifluorotoluene in chloroform-D). ^19^F NMR chemical shifts were calibrated to 0.1 M NaF in D_2_O (δ_F_ = −121.5 ppm) as an external standard. All NMR spectra were analyzed by MestReNova 12.0.1 ^19^F NMR parameters used for general compound characterization: ^19^F excitation pulse, 15.1 μs; acquisition time, 0.57 s; relaxation delay, 1.0 s; number of scans, 128; spectral width, 240 ppm; and spectral resolution, 0.83 Hz.

### ^19^F NMR Parameters Used for Enzymatic Experiments

^19^F excitation pulse, 15.1 μs; acquisition time, 0.57 s; relaxation delay, 1.0 s; number of scans, 128; spectral width, 15 ppm; transmitter frequency offset, −52.5 ppm; and spectral resolution, 1.72 Hz.

### Real-Time Experiments (Compounds **1** and **3**)

Before each experiment, the sample without the enzyme was incubated inside a magnet at 37 °C for 5 min and then locked, tuned, and shimmed, and initial 128 scans were recorded. After addition of protein, the spectra were recorded with fixed delays (300 s), and the number of experiments was set to 10. Conditions for the AtSOT18 protein were as follows: for **1** and **3**: 200 μM PAPS analogue, 200 μM desulfosinigrin, 100 nM enzyme, 83 mM Tris buffer, pH 9.0, 9.2 mM MgCl_2_, and 10% D_2_O, 37 °C; conditions for the SULT1A3 protein were as follows: 200 μM 2-F-PAPS (**1**), 200 μM dopamine, 100 nM enzyme, 6.7 mM K_2_HPO_4_, pH 6.5, and 10% D_2_O at 37 °C; or 200 μM 8-CF_3_-PAPS (**3**), 200 μM dopamine, 100 nM enzyme, 6.7 mM K_2_HPO_4_, pH 7.4, and 10% D_2_O at 37 °C.

### End-Point Assay (Compound **2**)

Conditions for AtSOT18:^14^ 200 μM 2-CF_3_-PAPS (**2**), 200 μM desulfosinigrin, 100 nM enzyme, 83 mM Tris buffer, pH 9.0, 9.2 mM MgCl_2_, and 10% D_2_O (275 μL in total). The reaction mixture was placed in a thermoblock at 37 °C, 300 rpm for 40 min. After this time, the reaction was terminated by the addition of acetonitrile (1:1; v/v), centrifuged, transferred to an NMR tube, and analyzed by ^19^F NMR; conditions for SULT1A3:^15^ 200 μM 2-CF_3_-PAPS (**2**), 200 μM dopamine, 100 nM enzyme, 6.7 mM K_2_HPO_4_, pH 7.4, and 10% D_2_O (200 μL in total). The reaction mixture was placed at 37 °C, 300 rpm for 40 min. After this time, the reaction was stopped by addition of acetonitrile (200 μL), adjusted to pH 8.5 by the addition of the mixture of 30 mM K_2_HPO_4_ buffer, pH 9.5, and 10% D_2_O (200 μL in total), centrifuged, transferred to an NMR tube, and analyzed by ^19^F NMR.

### Stability Experiments

Before each experiment, the sample without the enzyme was incubated inside a magnet at 37 °C for 5 min and then locked, tuned, and shimmed, and initial 128 scans were recorded. Each stability experiment was recorded with a fixed delay of 1800 s, and the number of experiments was set to 35.

### pH-Dependent Titrations

The mixtures of PAPS (**1**–**3**) and corresponding PAP analogues were prepared as follows: 10 mM solution of each PAPS analogue in H_2_O was adjusted to pH 3. The samples were incubated for ∼20 h at 37 °C, and progress of PAPS to PAP conversion was monitored by RP HPLC. The hydrolysis was continued until RP HPLC revealed formation of a non-equimolar mixture of PAPS/PAP with at least a 20% content of PAP. The samples for titration experiments were prepared as follows: the PAP and PAPS mixture generated by acidic hydrolysis was diluted to 200 μM (total concentration of nucleotides) in 6.7 mM K_2_HPO_4_ buffer containing 10% D_2_O, and pH of the resulting solutions was adjusted to 4.0. The solutions were centrifuged and transferred to NMR tubes, incubated inside a magnet at 37 °C for 5 min and then locked, tuned, and shimmed, and initial 128 scans were recorded. The samples were titrated by the addition of aq. NaOH (0.1 M), and pH was determined directly in the NMR tube (Extended Length pH Electrode with Micro Bulb, Hanna Instruments). ^19^F NMR spectra were recorded between pH 4.0–9.0 at approx. 0.5 unit steps. Each titration was performed in duplicate.

### Screening for SULT1A3

Enzymatic reactions for the screening of the LOPAC1280 library were performed in 96-well deep-well plates (BrandTech 701352 Deep-Well Plate, 96-well, PS, 1.1 mL, Standard, U-Bottom). Each well contained 200 μM 8-CF_3_-PAPS (**3**), 200 μM dopamine, the 35 μM tested inhibitor candidate (0.96 μL of the 10 mM stock solution), and 100 nM protein in 6.7 mM K_2_HPO_4_, pH 7.4, with addition of 10% D_2_O (275 μL in total). The control reaction without the inhibitor (performed in triplicate) included 35 μM (0.96 μL) DMSO instead of the inhibitor. The control reaction without protein (performed in triplicate) included water instead of the enzyme. The reactions were carried out at 37 °C for 40 min with mixing (300 rpm). After this time, each reaction was quenched by adding acetonitrile (275 μL) followed by 10 μL of buffered EDTA solution (20 mg mL^–1^ EDTA, 10 mg/mL^–1+^ NaHCO_3_) and was centrifuged, transferred to NMR tubes, and analyzed by ^19^F NMR.

## Supporting Information Available

AtSOT18 and SULT1A3 expression and purification procedures, detailed synthetic procedures, additional analytical data, and HRMS and NMR spectra of the final products (PDF)
