# Supplementary material for: Fluorinated Phosphoadenosine 5′-Phosphosulfate Analogues for Continuous Sulfotransferase Activity Monitoring and Inhibitor Screening by 19F NMR Spectroscopy
Source: ACS Chem Biol. 2022 Feb 23;17(3):661–9. doi: 10.1021/acschembio.1c00978 (PMC8938925; doi:10.1021/acschembio.1c00978)
Supplement: Supplementary file 1 — cb1c00978_si_001.pdf [file cb1c00978_si_001.pdf]

Supporting Information

Fluorinated PAPS analogs for continuous sulfotransferase activity monitoring and inhibitor screening by ^{19}F NMR spectroscopy

Agnieszka Mlynarska-Cieslak[†], Mikolaj Chrominski[‡], Tomasz Spiewla[‡], Marek R. Baranowski[†], Marcelina Bednarczyk[†,‡], Jacek Jemielity[‡], Joanna Kowalska[†,*]

[†]*Division of Biophysics, Institute of Experimental Physics, Faculty of Physics, University of Warsaw, Pasteura 5, 02-093 Warsaw, Poland*

[‡]*Centre of New Technologies University of Warsaw, Banacha 2c, 02-097 Warsaw, Poland*

**E-mail: jkowalska@fuw.edu.pl*

Table of Contents

Table of Contents 2
1. Supporting Tables 3
2. Supporting Figures 7
3. Experimental Procedures 11
3.1. AtSOT18 and SULT1A3 expression and purification 11
3.2. General Information for the chemical syntheses 12
3.3. Synthetic procedures 13
3.3.1. 2’,3’-cyclophospho-2-(fluoro)adenosine 5’-phosphorimidazolide (5a) 13
3.3.2. 2’,3’-cyclophospho-2-(trifluoromethyl)adenosine 5’-phosphorimidazolide (5b) 13
3.3.3. 2’,3’-cyclophospho-8-(trifluoromethyl)adenosine 5’-phosphaorimidazolide (5c) 13
3.4. General procedure A: Preparation of 3’-phosphoadenosine 5’-phosphosulfate derivatives 13
3.4.1. 3’-phospho-2-(fluoro)adenosine 5’-phosphosulfate (1) 14
3.4.2. 3’-phospho-2-(trifluoromethyl) adenosine 5’-phosphosulfate (2) 14
3.4.3. 3’-phospho-8-(trifluoromethyl) adenosine 5’-phosphosulfate (3) 14
3.5. HPLC assay 15
3.5.1. AtSOT18 15
3.5.2. SULT1A3 15
4. Raw data: HPLC profiles, NMR and HRMS spectra 16

1. Supporting Tables

Table S1. Library of compounds used for ^{19}F NMR screening with 8-CF_3-PAPS and SULT1A_3 and the determined % of inhibition values. The data are mean values from two experiments ± SD.

Compound symbol	Compound Name	% inhibition
P1B5	Aminophyline ethylenediamine	-6.57 ± 6.77
P2A8	1-Allyl-3,7-dimethyl-8-p-sulfophenylxanthine	-2.64 ± 7.52
P2D9	N6-2-(4-Aminophenyl)ethyladenosine	-0.51 ± 8.75
P2E8	Alaproclate hydrochloride	-4.24 ± 6.00
P2F6	8-(p-Sulfophenyl)theophylline	70.23 ± 41.47
P2G6	1,3-Dipropyl-8-p-sulfophenylxanthine	-0.45 ± 0.63
P2G9	AB-MECA	2.25 ± 0.03
P2H9	Alloxazine	10.04 ± 21.15
P3G9	Caffeine	0.89 ± 1.26
P4F5	2-Chloroadenosine	2.90 ± 13.57
P4G6	7-Chloro-4-hydroxy-2-phenyl-1,8-naphthyridine	26.28 ± 11.45
P4G9	N6-Cyclopentyladenosine	5.40 ± 1.21
P5A4	CGS-21680 hydrochloride	12.22 ± 9.24
P5A5	CGS-15943	58.69 ± 15.50
P5D7	L-3,4-Dihydroxyphenylalanine methyl ester hydrochloride	-2.44 ± 6.66
P5E10	Dilazep hydrochloride	-2.57 ± 6.77
P5G2	8-Cyclopentyl-1,3-dipropylxanthine	-2.57 ± 6.77
P5G10	1,7-Dimethylxanthine	-2.57 ± 6.77
P5G11	N-Methyldopamine hydrochloride	-2.57 ± 6.77
P5H4	8-(3-Chlorostyryl)caffeine	-1.72 ± 12.08

P6B6	6,7-ADTN hydrobromide	-25.97 ± 41.55
P6B8	1,3-Dipropyl-7-methylxanthine	1.79 ± 15.15
P6C9	3,7-Dimethyl-1-propargylxanthine	4.91 ± 10.73
P6D11	Etazolate hydrochloride	-10.33 ± 22.64
P6F5	L-DOPA	3.13 ± 13.26
P6G5	Dipyridamole	21.55 ± 28.87
P6G6	Dipropyldopamine hydrobromide	-8.20 ± 3.55
P6G7	Naratriptan hydrochloride	-3.25 ± 2.98
P6G11	5’-N-Ethylcarboxamidoadenosine	-4.95 ± 18.25
P7A5	S-(-)-Eticlopride hydrochloride	21.31 ± 14.06
P7C5	erythro-9-(2-hydroxy-3-nonyl)adenine hydrochloride	22.87 ± 16.27
P7E7	Fusaric acid	-0.63 ± 7.32
P8C7	Dopamine hydrochloride	-36.79 ± 23.10
P8H4	6-Hydroxy-DL-DOPA	95.25 ± 2.93
P9A3	3-Isobutyl-1-methylxanthine	4.67 ± 7.86
P9D6	IB-MECA	-3.02 ± 15.01
P10D2	cis(+/-)-8-OH-PBZI hydrobromide	-5.22 ± 12.20
P10D10	alpha,beta-Methylene adenosine 5’-triphosphate dilithium	3.59 ± 1.87
P10E3	Metoclopramide hydrochloride	8.73 ± 9.13
P10G4	MRS 1523	13.76 ± 14.64
P11D7	S-(4-Nitrobenzyl)-6-thioinosine	5.48 ± 6.14
P12C3	CP-66713	-0.55 ± 2.38
P12C11	3-n-Propylxanthine	-1.87 ± 10.22

P12D8	1,3-Dimethyl-8-phenylxanthine	3.75 ± 5.94
P12H9	R(-)-N6-(2-Phenylisopropyl)adenosine	9.13 ± 1.55
P13A9	Pregnenolone sulfate sodium	19.78 ± 5.48
P13B7	2-Phenylaminoadenosine	1.95 ± 5.28
P13D7	S(-)-3PPP hydrochloride	2.29 ± 28.96
P13F11	Quinelorane dihydrochloride	-6.76 ± 17.59
P13H9	Pramipexole dihydrochloride	-0.79 ± 0.49
P14E10	(-)-Sulpiride	-1.79 ± 15.15
P14H6	Sulindac sulfone	5.19 ± 2.30
P15B6	Theophylline	-0.32 ± 19.74
P15G8	Theobromine	2.60 ± 18.83
P16F10	SCH 58261	-5.54 ± 14.26
P16E11	Zonisamide sodium	3.51 ± 20.75
ADP	adenosine 5’-diphosphate	4.40 ± 19.49
ATP	adenosine 5’-triphosphate	6.92 ± 7.89
PAP	3’-phospho adenosine 5’-phosphate	70.62 ± 16.30

2. Supporting Figures

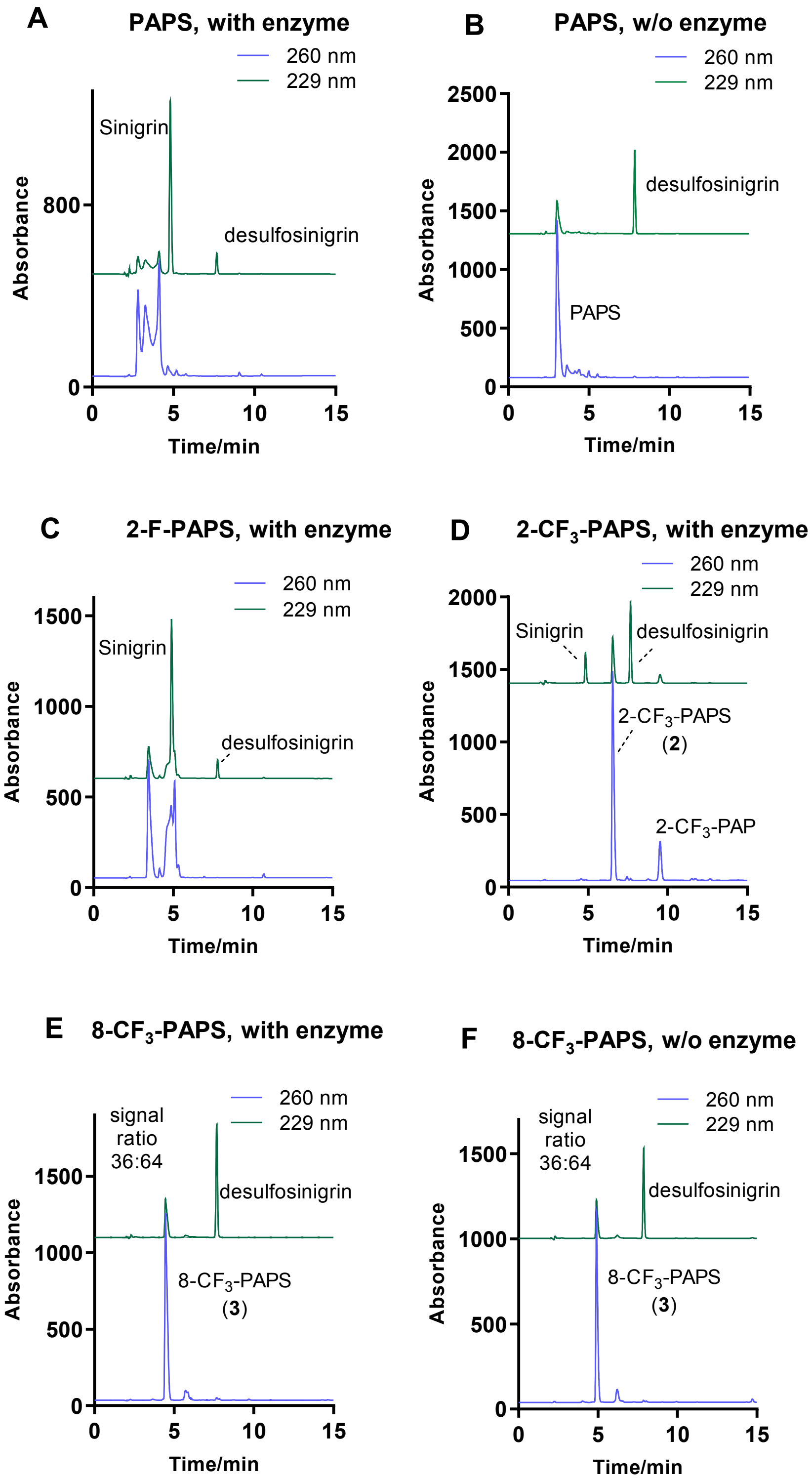

Figure S1. RP HPLC analysis of AtSOT18-catalyzed desulfosinigrin sulfation reactions performed in the presence of: A) PAPS; C) 2-F-PAPS; D) 2-CF_3-PAPS; E) 8-CF_3-PAPS; B and F) control samples without AtSOT18 for PAPS and 8-CF_3-PAPS shown to facilitate interpretation of the results.

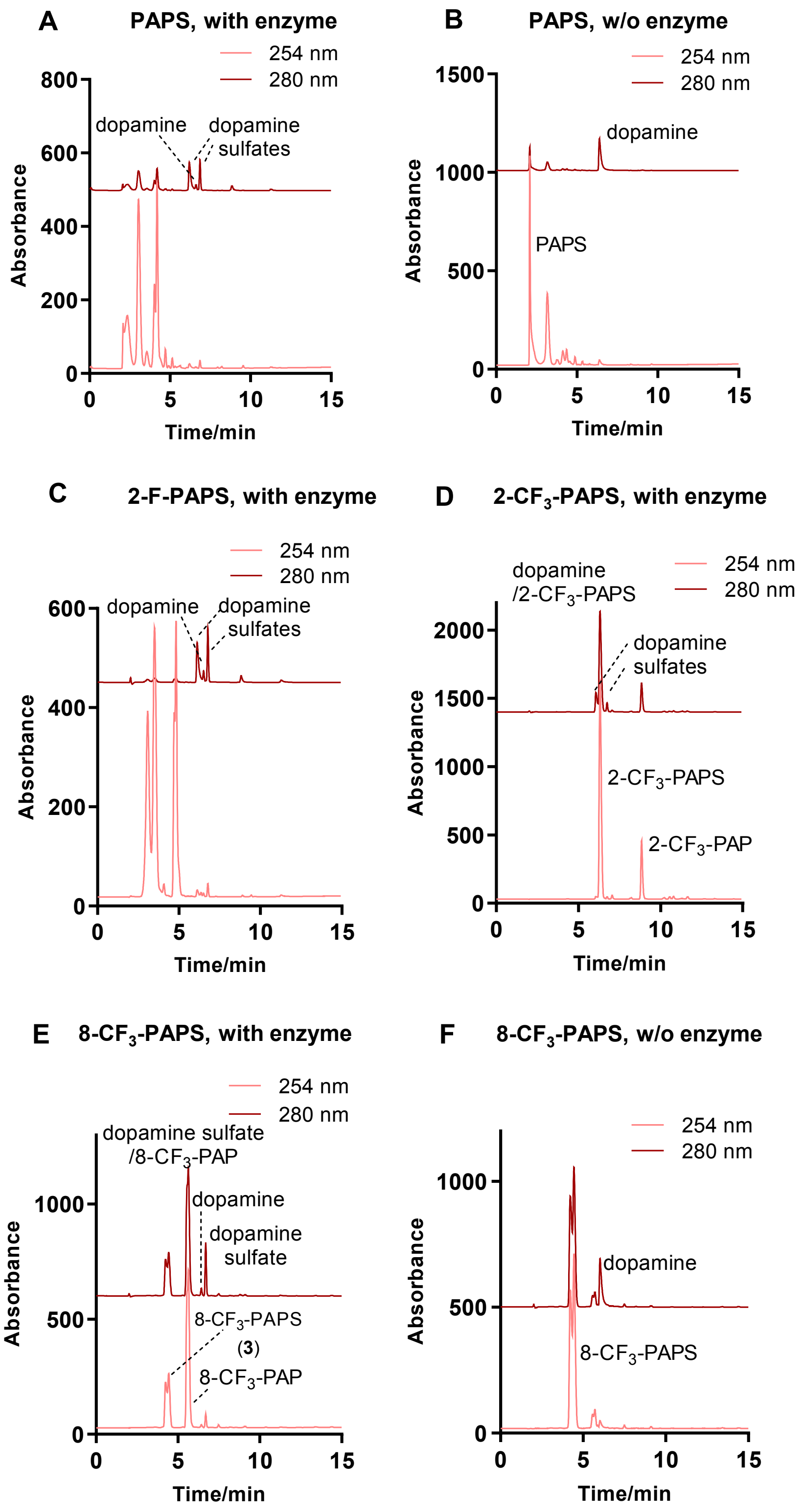

Figure S2. RP HPLC analysis of SULT1A3-catalyzed dopamine sulfation reactions performed in the presence of: A) PAPS; C) 2-F-PAPS; D) 2-CF_3-PAPS; E) 8-CF_3-PAPS; B and F) control samples without SULT1A3 shown to facilitate interpretation of the results.

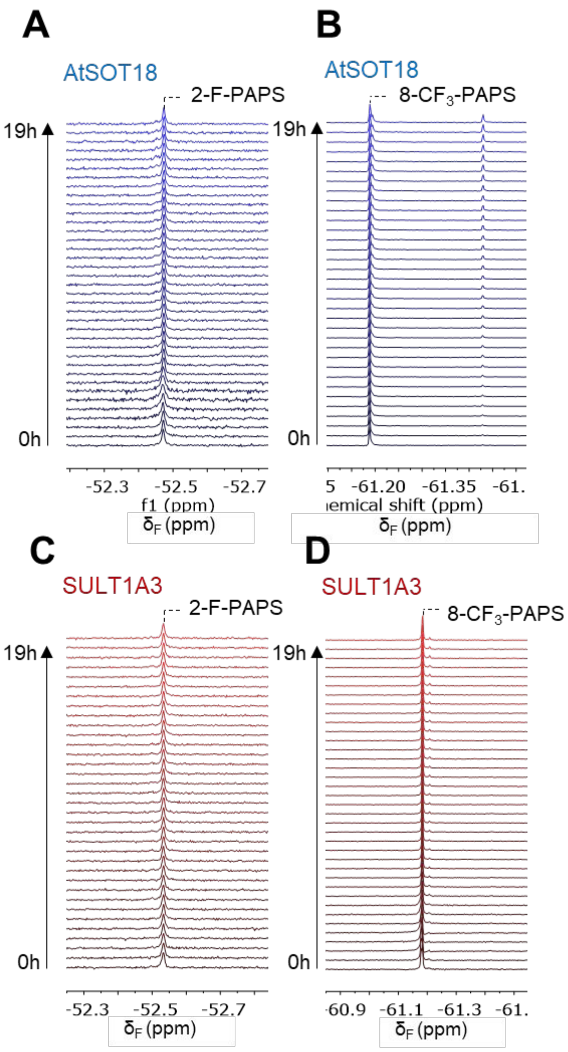

Figure S3. ^{19}F NMR-monitored analysis of 2-F-PAPS (A,C) and 8-CF_3-PAPS (B,D) stability in AtSOT18 (A,B) and SULT1A3 (C,D) buffers. Conditions for AtSOT18 (A,B): 200 µM PAPS analog, 100 µM desulfosinigrin, 83 mM Tris buffer, pH 9.0, 9.2 mM $MgCl_2$, 10% D_2O, 37 °C; for SULT1A3 (C,D): 200 µM PAPS analog, 100 µM dopamine, 6.7 mM K_2HPO_4, pH 6.5 (for 2-F-PAPS) or 7.4 (for 8-CF_3-PAPS), 10% D_2O at 37 °C. The product of 8-CF_3-PAPS decomposition at pH 9.0 (δ_F -61.43) was identified as 8-CF_3-adenine.

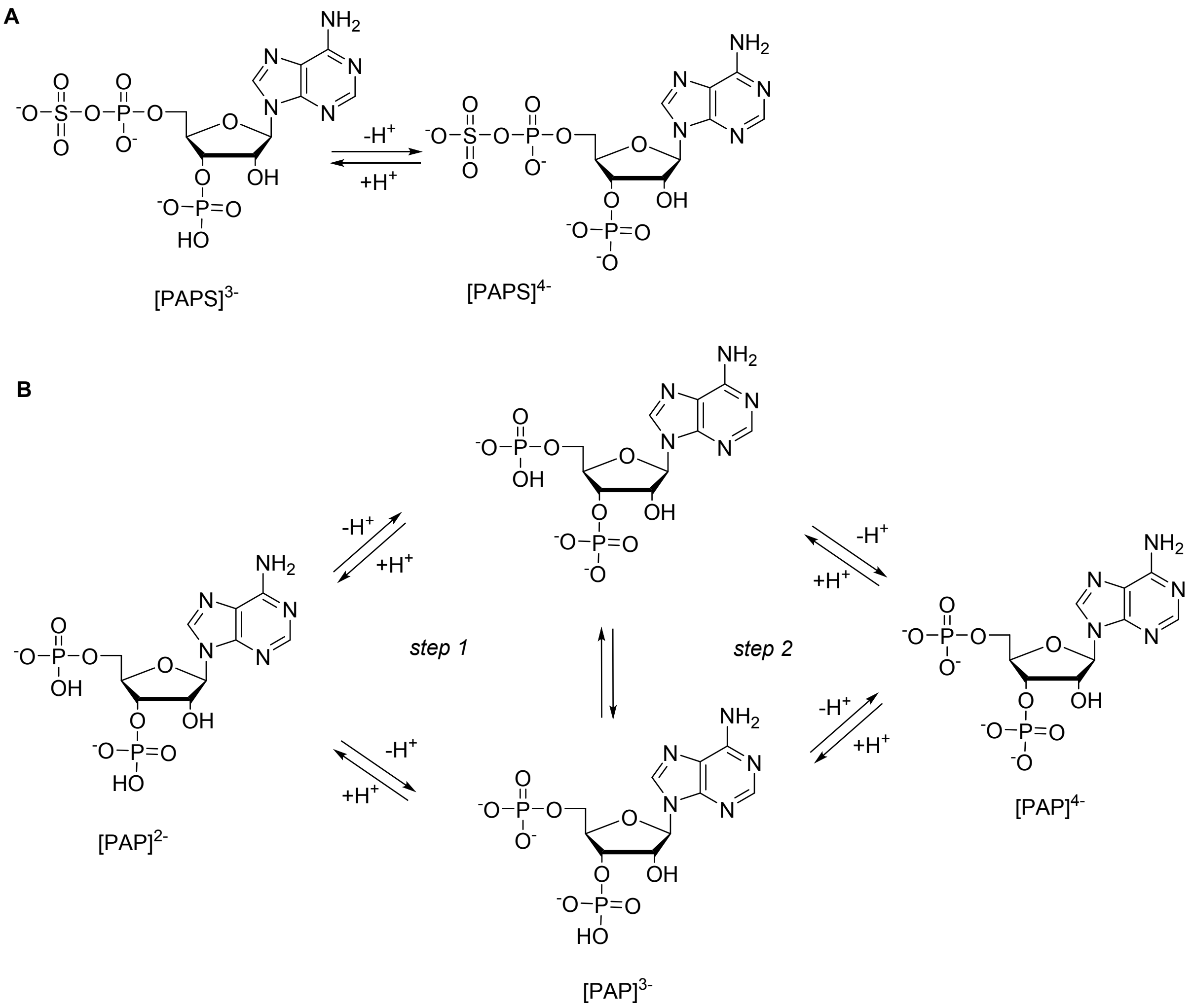

Figure S4. Acid-base equilibria at pH values close to physiological for A) PAPS; B) PAP. Probably, all four microscopic acidic dissociation equilibria shown in B contribute to the macroscopic *p*Ka of PAP determined by ^{19}F NMR.

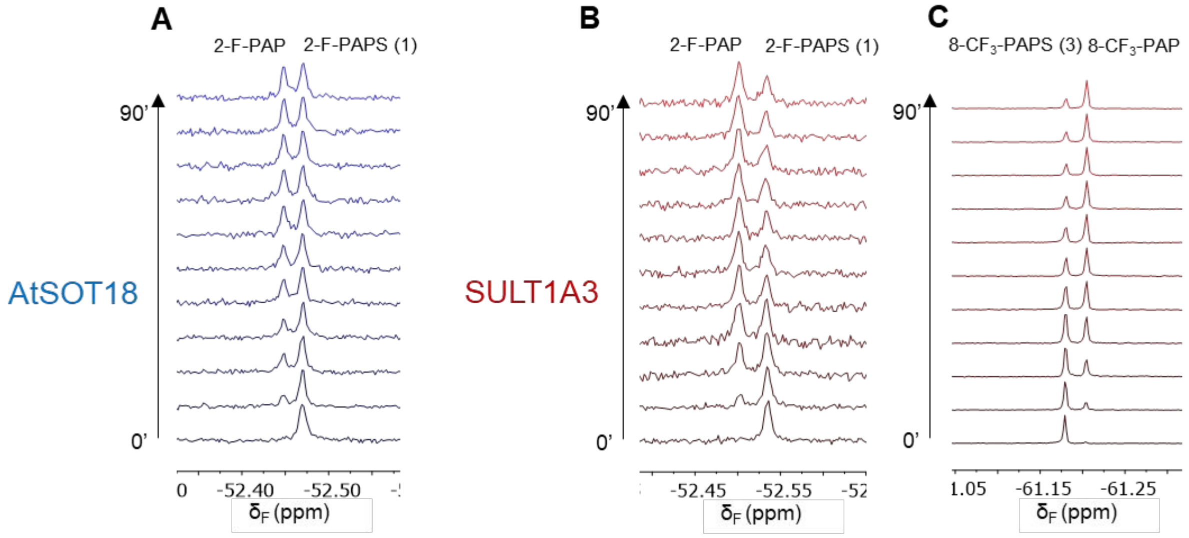

Figure S5. ^{19}F NMR real-time monitoring of sulfotransferase activity under optimized assay conditions. A) AtSOT18 in the presence of 2-F-PAPS; B) SULT1A3 and 2-F-PAPS; C) SULT1A3 and 8-CF_3-PAPS. Conditions are given in Table 1 in the main manuscript and in the Experimental Section below.

3. Experimental Procedures

3.1. AtSOT18 and SULT1A3 expression and purification

The sequence encoding SOT18 from Arabidopsis thaliana (Gene ID- 834749) in plasmid vector (pQE-30_His_AtSOT18) was obtained courtesy of Jutta Pappenbrock and Felix Hirschmann[1]. AtSOT18 protein (~42 kDa) flanked with His-tag on its N1-end was overexpressed in BL21 (DE3) RIL E.coli (Invitrogen) Prokaryotic system. 6xHis-AtSOT18 sulfotransferase expression was induced by 0.4 mM IPTG (Isopropyl-β-D-thiogalactoside) at optical density of 0.7 (bacterial culture) and the cells were further cultured for 16 h at 18°C. The cells were harvested and lysed in a buffer containing: 50 mM Na_2HPO_4 (pH 7.5), 300 mM NaCl, 30 mM imidazole, 0.1 mg/ml lysozyme and mixture of protease inhibitors (Aprotinin, Leupeptiun, Pepstatin, PMSF). The lysate was sonicated (15 min, Amplitude 50%, 15 s on/off) and centrifuged. The supernatant was then loaded on 2 x 5 ml HisTrap FFTM column (Cytiva) previously equilibrated with a buffer containing 50 mM Na_2HPO_4 (pH= 7.5), 300 mM NaCl, 30 mM imidazole. Protein AtSOT18 was eluted with a buffer containing 50 mM Na_2HPO_4 (pH= 7.5), 300 mM NaCl, 500 mM imidazole. The collected fractions were further purified on a Superdex 75 pg HiLoad 26/600 gel filtration column (Cytiva). Samples with 6xHis-AtSOT18 protein were concentrated to 30 µM, flash frozen and stored at -80 °C in a buffer containing 20 mM Tris-HCl (pH 8.0), 100 mM NaCl, 1 mM DTT, 10% glycerol.

The human sulfotransferase SULT1A3 (Gene ID- 6818) sequence in plasmid vector (pUC57-SULT1A3) was purchased from GenScript and cloned into the pET28 vector. The 6xHis-SULT1A3 cDNA was amplified by PCR using the sense primer 5'-TTAGGATCCATGGAACTGATTCAAGACACCAG-3' which inserts a BamHI site and the antisense primer 5'-GAGAAGCTTTTACAGTTCGCTACGAAAGCTCAG-3' which inserts a HindIII restriction site and plasmid pUC57-SULT1A3 as a template. SULT1A3 insert and vector pET28 were digested with FD BamHI (Thermo Scietific) and FD HindIII (Thermo Scietific) enzymes, then ligated and transformed into *E.coli* Top10 (Invitrogen). Restriction analysis and sequencing were used to confirm the correct sequence gene in the target vector (pET28-His-SULT1A3). Overexpression and purification of sulfotransferase SULT1A3 (~36 kDa) was conducted analogously to AtSOT18. Harvested cells were lysed in a buffer containing: 50 mM HEPES (pH 7.5), 300 mM NaCl, 30 mM imidazole, 5 mM ß-merkaptoetanol, 0.1 mg/ml lysozyme, and mixture of protease inhibitors. The lysate was sonicated, centrifuged and loaded on 2 x 5 ml HisTrap FFTM column (Cytiva) previously equilibrated with a lysis buffer (without lysozyme and protease inhibitors). 6xHis-SULT1A3 protein was eluted with a buffer containing 300 mM imidazole. The final purification step was gel filtration on a Superdex 75 pg HiLoad 26/600 gel filtration column (Cytiva). Fractions with 6xHis-SULT1A3 protein concentrated to 30 µM, flash frozen and stored at -80 °C in a buffer containing 50 mM HEPES (pH 7.5), 100 mM NaCl, 1 mM DTT, 10% glycerol. The homogeneity of both protein was confirmed by SDS-PAGE electrophoresis (Figure S6).

`

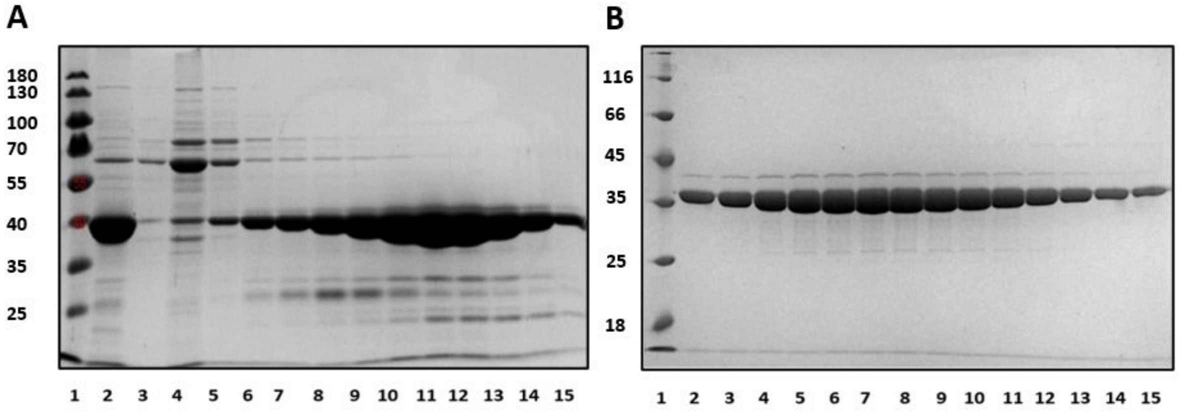

Figure S6. Analysis of protein purity. Purified proteins AtSOT18 (A) and SULT1A3 (B) were analysed by SDS-PAGE. A) Polyacrylamide gel foe protein AtSOT18 after gel filtration (Superdex 75 pg 26/600), signal at level ~ 42 kDa, fractions 6-15; B) Polyacrylamide gel for protein SULT1A3

after gel filtration (Superdex 75 pg 26/600), signal at level ~ 36 kDa, fraction 2-15 A-PageRuler™ Prestained Protein Ladder (26617), B-Pierce™Unstained Protein MW Marker (26610).

3.2. General Information for the chemical syntheses

Solvents and chemical reagents were acquired from commercial sources. 2-fluoro adenosine was purchased from Sigma Aldrich. RNAse T2 was purchased from MoBiTec (Germany). 8-trifluoromethyl adenosine and 2-trifluoromethyl adenosine were synthesized as described by Chrominski et al.[2]

The purification of imidazole derivatives and final compounds was performed by ion-exchange chromatography on a DEAE Sephadex A-25 (HCO_3^- form) column. To this end, the column was loaded with the reaction mixture and washed thoroughly with water until the eluate did not precipitate with $AgNO_3$ solution (to remove residual solvents and non-binding reagents). Nucleotides were eluted using a 0.8 M and 1 M linear gradient of triethylammonium bicarbonate (TEAB) in deionized water for imidazole derivatives and final compounds respectively. Collected fractions were analyzed spectrophotometrically at 260 nm and by RP-HPLC. Fractions containing the desired products **6a-c** were combined and concentrated under reduced pressure with repeated additions of ethanol (96%) and acetonitrile, to decompose TEAB and to remove residual water, respectively. Compounds **6a-c** were isolated as triethylammonium salts. Yields were calculated on the basis of optical density units (OD = volume in mL × absorbance of the solution) of the isolated products and corresponding starting materials (nucleotides or nucleotide *P*-imidazolide derivatives), except when described otherwise. The extinction coefficients for **4a** was the same as for non-modified adenine, 15020 (ε, mL/mmol/cm), for **4b** and **4c** were determined by diluting a specified mass of compounds in DMSO and measuring absorbance of the solution at 260 nm in 0.1 M phosphate buffer, pH 7.0, at room temperature. The extinction coefficients (ε_{260}) determined for **4b** and **4c** were 10660 and 11392 (ε, mL/mmol/cm), respectively. It was assumed that the extinction coefficients for nucleotides **1-3** derived from **4a–c** are the same as those of the parent nucleosides.

The semi-preparative RP HPLC purification of compounds **6a-c** was performed using a Clarity 5 µm Oligo-RP LC column (150×10 mm, flow rate 5.0 mL min^{-1}) with UV detection at 254 nm and isocratic gradient (for **6a**) of buffer A (0.05 M triethylammonium acetate buffer, pH 7) or linear gradient from buffer A to 50% of buffer B (1:1 of ACN : 0.1 M buffer A) over 60 min (for **6b** and **6c**). After repeated freeze-drying of the collected fractions, products were isolated as triethylammonium salts.

Analytical RP HPLC (for chemical reaction progress monitoring and enzymatic assays; Figure 2I,J; Figure 1S, Figure 2S) was performed using Gemini 3 µm NX-C18 LC column 110 Å (150×4.6 mm, flow rate 1 mL min^{-1}). A linear gradient from buffer A (ammonium acetate buffer, pH 5.9, 0.05 M) to 50% of buffer B (50:50 of methanol:buffer A) over 15 min was applied for compound **1** or to 100% of buffer B for **2** and **3**.

The structures and homogeneities of all final compounds were confirmed by RP HPLC, high-resolution mass spectrometry using negative electrospray ionization (HRMS-ESI) and ^{1}H, ^{31}P and ^{19}F NMR spectroscopy. Mass spectra were recorded with LTQ Orbitrap Velos (Thermo Scientific) spectrometer. NMR spectra were recorded at 25 °C with a BRUKER AVANCE III HD spectrometer at 500 MHz (^{1}H NMR), 202 MHz (^{31}P NMR) and 471 MHz (^{19}F NMR). ^{1}H NMR chemical shifts were calibrated to sodium 3-trimethylsilyl-[2,2,3,3-D4]propionate (TSP) in D_2O, for ^{31}P NMR to H_3PO_4 (20%) in D_2O and for ^{19}F NMR to 0.1 M NaF in D_2O (δF = −121.5 ppm) as an external standard. Signal assignments and identification were based on COSY spectra analysis. The NMR spectra were analyzed by MestReNova 12.0.1.

3.3. Synthetic procedures

3.3.1. 2',3'-cyclophospho-2-(fluoro)adenosine 5'-phosphorimidazolide (5a)

To the solution of 2-fluoroadenosine (**4a**;110 mg, 0.39 mmol) in trimethyl phosphate (2 mL) $POCl_3$ (177 mg, 1.16 mmol, 3 equiv.) was added at 0°C (ice bath) and stirred for 3 h. After that time, a second portion of $POCl_3$ (354 mg, 2.31

mmol, 6 equiv.) was added. The mixture was left overnight at 0 °C and allowed to warm up to 24 °C and then poured into cold diethyl ether and centrifuged. Supernatant was discarded and the precipitate was diluted in trimethyl phosphate (2 mL) followed by addition of imidazole (1414 mg, 20.80 mmol, 54 equiv.). After 1 h the reaction was quenched with 10 volumes of water and purified by DEAE-Sephadex using a linear gradient of TEAB (0–0.8 M), to obtain the product as triethylammonium salt (1644 OD, 0.11 mmol, 28% yield).

3.3.2. 2',3'-cyclophospho-2-(trifluoromethyl)adenosine 5'-phosphorimidazolide (5b)

To the solution of 2-(trifluoromethyl)adenosine (**4b**; 49 mg, 0.15 mmol) in trimethyl phosphate (1 mL), containing 2.6-lutidine (47 mg, 0.44 mmol, 3 equiv.), $POCl_3$ (67 mg, 0.44 mmol, 3 equiv.) was added at -5°C and stirred for 4 h. After that time, a second portion of $POCl_3$ (134 mg, 0.87 mmol, 6 equiv.) was added. The mixture was left overnight at - 5°C and then poured into cold diethyl ether and centrifuged. Supernatant was discarded and the precipitate was diluted in trimethyl phosphate (2 mL) followed by addition of imidazole (534 mg, 7.85 mmol, 54 equiv.). After 1 h reaction was quenched with 10 volumes of water and purified by DEAE-Sephadex using a linear gradient of TEAB (0 – 0.8 M), to obtain the product as triethylammonium salt (163 mOD, 0.02 mmol, 11% yield).

3.3.3. 2',3'-cyclophospho-8-(trifluoromethyl)adenosine 5'-phosphaorimidazolide (5c)

To the solution of 8-(trifluoromethyl)adenosine (**4c**; 101 mg, 0.30 mmol) in trimethyl phosphate (2 mL) and 2.6-lutidine (96 mg, 0.90 mmol, 3 equiv.) $POCl_3$ (138 mg, 0.90 mmol, 3 equiv.) was added at 0°C and stirred for 3 h. After that time, a second portion of $POCl_3$ (276 mg, 1.80 mmol, 6 equiv.) was added. The mixture was left overnight at 0°C and allowed to warm up to 24°C and then poured into cold diethyl ether and centrifuged. Supernatant was discarded and the precipitate was diluted in trimethyl phosphate (2 mL) followed by addition of imidazole (1101 mg, 16.20 mmol, 54 equiv.). After 1 h reaction was quenched with 10 volumes of water and purified by DEAE-Sephadex using a linear gradient of TEAB (0 – 0.8 M), to obtain the product as triethylammonium salt (495 mOD, 0.04 mmol, 14% yield).

3.4. General procedure A: Preparation of 3'-phosphoadenosine 5'-phosphosulfate derivatives

2',3'-Cyclophosphoadenosine 5'-phosphorimidazolide derivative was diluted in DMF, and bis(tributylammonium) sulfate and anhydrous $MgCl_2$ were added. The reaction was stirred for at least 18 h (until total conversion of substrate was observed by RP-HPLC) and then quenched by addition of equimolar solution of EDTA in H_2O (the pH was adjusted to 7 by the addition of $NaHCO_3$).

3.4.1. 3'-phospho-2-(fluoro) adenosine 5'-phosphosulfate (1)

The synthesis was performed following the general procedure **A** using 2',3'-cyclophospho-2-fluoro adenosine 5'-phosphoimidazolide (**5a**; 63 mg, 0.11 mmol), DMF (2 mL), bis(tributylammonium) sulfate (204 mg, 0.43 mmol, 4 equiv.) and $MgCl_2$ (86 mg, 0.90 mmol, 8 equiv.). **2'3'-cyclophospho-2-(fluoro) adenosine 5'-phosphate (6a)** was purified by DEAE-Sephadex (yield: 1189 mOD, 0.08 mmol, 72%), followed by semi-preparative RP HPLC. Final yield: 28 mg, 0.41 mmol, 52%.

^{1}H NMR (500 MHz, D$_2$O): δ 8.36 (s, 1 H, H_A), 6.23 (d, $J_{H,H}$ 4.2 Hz, 1 H, H1′), 5.41 (ddd, $J_{H,H}$ 4.3, 6.7, 11.0 Hz, 1 H, H2′), 5.27 – 5.20 (m, 1 H, H3′), 4.66– 4.61 (m, 1 H, H4′), 4.31 – 4.23 (m, 2 H, H5′, H5″) ppm, **^{31}P NMR (202 MHz, D$_2$O):** δ 20.50 (d, $J_{P,H}$ 7.4, 11.0 Hz, 1 P, P_{cyclic}), -9.98 – -10.15 (m, 1 P, P5') ppm, **^{19}F NMR (471 MHz, D$_2$O):** δ - 52.46 (s, 1 F, 2 F_A) ppm.

Enzymatic cleavage of compound **1** (10 mg/mL) was performed with RNAse T2 (750 U, 5 µL/10 mg, MoBiTec) in 50 mM ammonium acetate buffer, pH 7.3. The reaction mixture was placed in thermoblock at 37°C, 300 rpm. The reaction progress was monitored by RP HPLC. After 24 h the reaction was filtered through 10 K Amicon Ultra-0.5 mL Centrifugal Filters. The concentrate was discarded and the flow-through was used as a stock solution of **1** for NMR experiments (recovery greater than 90%).

HR-MS (ESI, *m/z*): Calculated for $C_{10}H_{13}FN_5O_{13}P_2S^-$ ([M-H]$^-$): 523.96953; Found: 523.96991.

^{1}H NMR (500 MHz, D_2O): δ 8.49 (s, 1 H, H_A), 6.05 (d, $J_{H,H}$ 6.9 Hz, 1 H, H1′), 4.85 – 4.72 (m, overlap with D_2O, 2 H, H2′, H3′), 4.60 – 4.57 (m, 1 H, H4′), 4.29 – 4.20 (m, 2 H, H5′, H5″) ppm, **^{31}P NMR (202 MHz, D_2O):** δ 3.64 – 3.29 (m, 1 H, P3'), -9.57 – -9.82 (m, 1 P, P5') ppm, **^{19}F NMR (471 MHz, D_2O):** δ - 52.95 (s, 1 F, 2 F_A) ppm.

3.4.2. 3'-phospho-2-(trifluoromethyl) adenosine 5'-phosphosulfate (2)

The synthesis was performed following the general procedure **A** using 2',3'-cyclophospho-2-trifluoromethyl adenosine 5'-phosphoimidazolide (**5b**; 10 mg, 0.02 mmol), DMF (0.5 mL), bis(tributylammonium) sulfate (17 mg, 0.04 mmol, 2 equiv.) and $MgCl_2$ (16 mg, 0.17 mmol, 11 equiv.). **2'3'-cyclophospho-2-(trifluoromethyl) adenosine 5'-phosphate (6b)** was purified by DEAE-Sephadex (yield: 68 mOD, 0.01 mmol, 42%), followed by semi-preparative RP HPLC. Final yield: 1.2 mg, 0.02 mmol, 27%.

^{1}H NMR (500 MHz, D_2O): δ 8.56 (s, 1 H, H_A), 6.44 (d, $J_{H,H}$ 3.9 Hz, 1 H, H1′), 5.49 (ddd, $J_{H,H}$ 3.9, 6.6, 10.4 Hz, 1 H, H2′), 5.31 (ddd, $J_{H,H}$ 3.6, 6.6, 8.0 Hz, 1 H, H3′), 4.71 – 4.64 (m, 1 H, H4′), 4.30 – 4.25 (m, 2 H, H5′, H5″) ppm, **^{31}P NMR (202 MHz, D2O):** δ 20.55 (dd, $J_{P,P}$ 8.0, 10.4 Hz, 1 P, P_{cyclic}), -9.85 – -10.14 (m, 1 P, P5') ppm, **^{19}F NMR (471 MHz, D_2O):** δ - 69.28 (s, 3 F, 2 F_A) ppm.

Enzymatic cleavage of compound **2** (10 mg/mL) was performed with RNAse T2 (750 U, 5 µL/10 mg, MoBiTec) in 50 mM ammonium acetate buffer, pH 7.3. The reaction mixture was placed on termomixer ar 37°C, 300 rpm. Progress of the reaction was monitored by RP HPLC. After 24 h the reaction was purified by 10 K Amicon Ultra-0.5 mL Centrifugal Filters (recovery greater than 90%).

HR-MS (ESI, *m/z*): Calculated for $C_{11}H_{13}F_3N_5O_{13}P_2S^-$ ($[M-H]^-$): 573.96634; Found: 573.96687.

^{1}H NMR (500 MHz, D_2O): δ 8.68 (s, 1 H, H_A), 6.27 (d, $J_{H,H}$ 6.7 Hz, 1 H, H1′), 4.89 – 4.82 (m, 2 H, H2′, H3′), 4.64 – 4.60 (m, 1 H, H4′), 4.29 – 4.25 (m, 2 H, H5′, H5″) ppm, **^{31}P NMR (202 MHz, D_2O):** δ 1.87 – 1.16 (m, 1 P, P3'), -9.61 – -10.17 (m, 1 P, P5') ppm, **^{19}F NMR (471 MHz, D_2O):** δ - 69.24 (s, 3 F, 2 CF_3) ppm.

3.4.3. 3'-phospho-8-(trifluoromethyl) adenosine 5'-phosphosulfate (3)

The synthesis was performed following the general procedure **A** using 2',3'-cyclophospho-8-trifluoromethyl adenosine 5'-phosphoimidazolide (**5c**; 27 mg, 0.04 mmol), DMF (2 mL), bis(tributylammonium) sulfate (83 mg, 0.18 mmol, 4 equiv.) and $MgCl_2$ (33 mg, 0.35 mmol, 8 equiv.). **2'3'-cyclophospho-8-(trifluoromethyl) adenosine 5'-phosphate (6c)** was purified by DEAE-Sephadex (yield: 278 mOD, 0.02 mmol, 56%), followed by semi-preparative RP HPLC. Final yield: 7 mg, 0.01 mmol, 43%.

^{1}H NMR (500 MHz, D_2O): δ 8.37 (s, 1 H, H_A), 6.41 (d, $J_{H,H}$ 3.0 Hz, 1 H, H1′), 5.81 – 5.75 (m, 1 H, H2′), 5.39 (ddd, $J_{H,H}$ 5.3, 7.1, 12.1 Hz, 1 H, H2′), 4.57 – 4.53 (m, 1 H, H4′), 4.34 – 4.18 (m, 2 H, H5′, H5″) ppm, **^{31}P NMR (202 MHz, D_2O):** δ 21.18 (dd, $J_{P,H}$ 7.1, 12.1 Hz, 1 P, P_{cyclic}), -9.89 (t, $J_{P,H}$ 6.2 Hz, 12.5, 1 P, P5') ppm, **^{19}F NMR (471 MHz, D_2O):** δ - 61.74 (s, 3 F, 8 CF_3) ppm.

Enzymatic cleavage of compound **3** (10 mg/mL) was performed with RNAse T2 (750 U, 5 µL/10 mg, MoBiTec) in 50 mM ammonium acetate buffer, pH 7.3. The reaction mixture was placed on termomixer ar 37°C, 300 rpm. Progress of the reaction was monitored by RP HPLC. After 24 h the reaction was purified by 10 K Amicon Ultra-0.5 mL Centrifugal Filters (recovery greater than 90%).

HR-MS (ESI, *m/z*): Calculated for $C_{11}H_{13}F_3N_5O_{13}P_2S^-$ ($[M-H]^-$): 573.96634; Found: 573.96678.

^{1}H NMR (500 MHz, D_2O): δ 8.38 (s, 1 H, H_A), 6.13 (d, $J_{H,H}$ 6.1 Hz, 1 H, H1′), 5.50 (t, $J_{H,H}$ 6.1 Hz, 1 H, H2′), 5.01 – 4.95 (m, 1 H, H3′), 4.56 – 4.51 (m, 1 H, H4′), 4.42 – 4.28 (m, 2 H, H5′, H5″) ppm, **^{31}P NMR (202 MHz, D_2O):** δ 1.04 (d, $J_{P,H}$ 8.8 Hz, 1 P, P3'), -9.89 (t, $J_{P,H}$ 6.2 Hz, 12.5, 1 P, P5')ppm, **^{19}F NMR (471 MHz, D_2O):** δ - 61.24 (s, 3 F, 8 CF_3) ppm.

3.5. HPLC assay

HPLC buffers:

A: 0.1 M KH_2PO_4/ K_2HPO_4 buffer, pH 6

B: buffer A: methanol 1:1

The experiments were analyzed using Supelcosil LC-18-T column (4.6 × 250 mm, 5 μM, flow rate 1.3 mL min^{-1}) with linear gradient from buffer A to 50% of buffer B in 15 min.

3.5.1. AtSOT18

Conditions: 200 μM of compound (**1**, **2** or **3**), 83 mM TRIS buffer, pH 9, 9.2 mM $MgCl_2$, 100 μM desulfosinigrin, 100 nM of protein, 37°C, 300 rpm,1 h.

The experiment was analyzed with absorbance detection at 260 nm and 229 nm.

3.5.2. SULT1A3

Conditions: 200 μM of compound (**1**, **2** or **3**), 6.7 mM K_2HPO_4 buffer, pH 7.4, 100 μM dopamine, 100 nM of protein, 37°C, 300 rpm,1 h.

The experiment was analyzed with absorbance detection at 254 nm and 280 nm.

References

[1] F. Hirschmann, J. Papenbrock, *Plant Physiology and Biochemistry* **2015**, *91*, 10-19.

[2] M. Chrominski, M. R. Baranowski, S. Chmielinski, J. Kowalska, J. Jemielity, *Journal of Organic Chemistry* **2020**, *85*, 3440-3453.

4. Raw data: HPLC profiles, NMR and HRMS spectra

Compound 1 : 2-F-PAPS	
Chemical structure	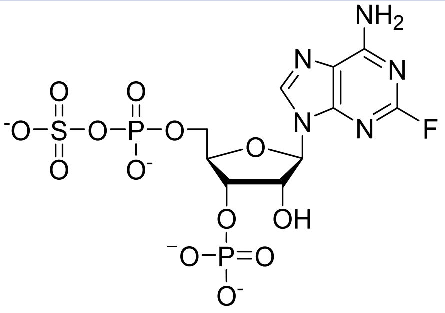
HPLC	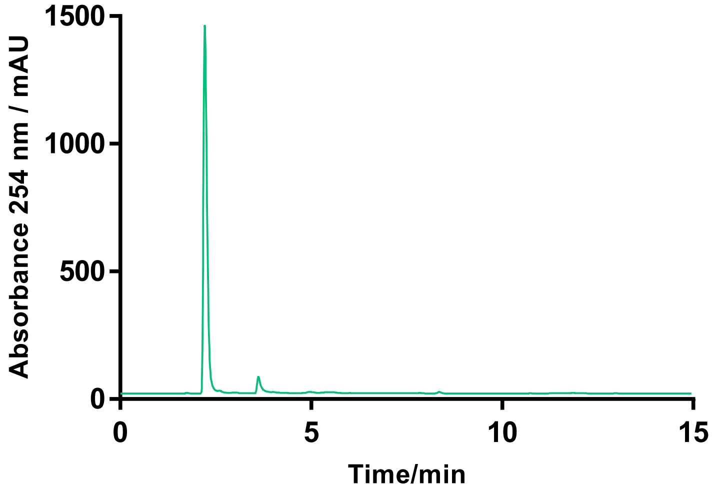

HRMS (-) ESI (Calc. [M-H]$^-$ $C_{10}H_{13}FN_5O_{13}P_2S^-$: 523.96953)

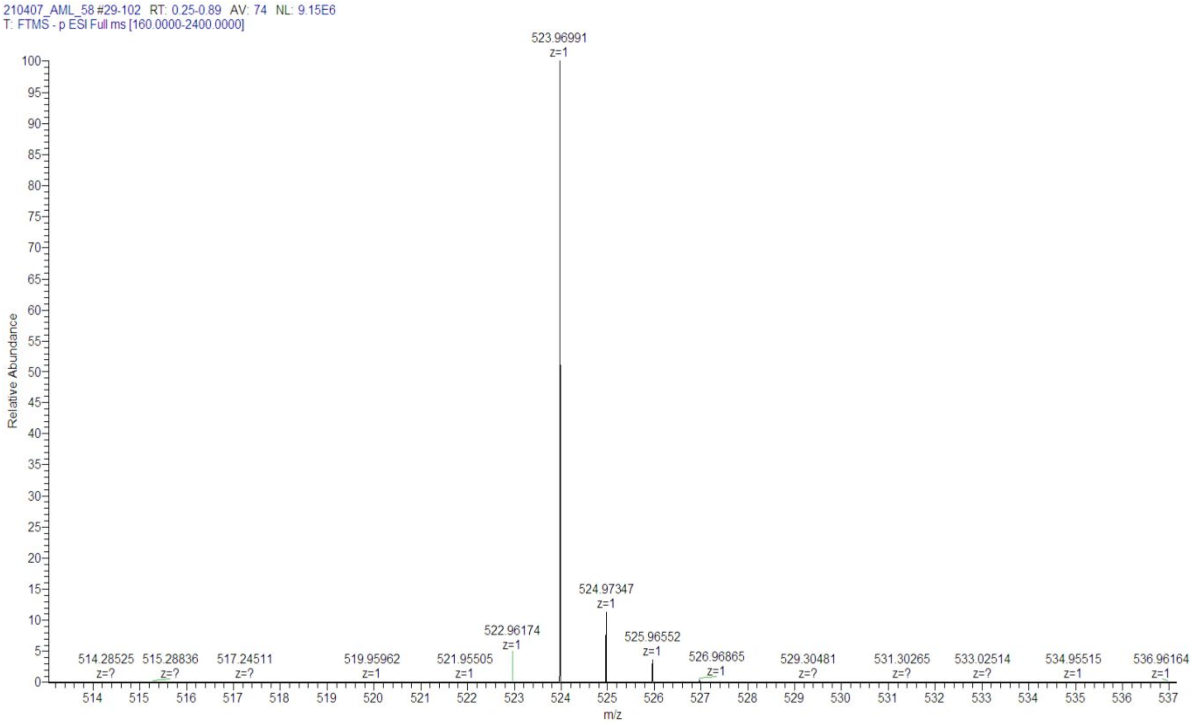

^{1}H NMR (500 MHz, D_2O, 25°C)

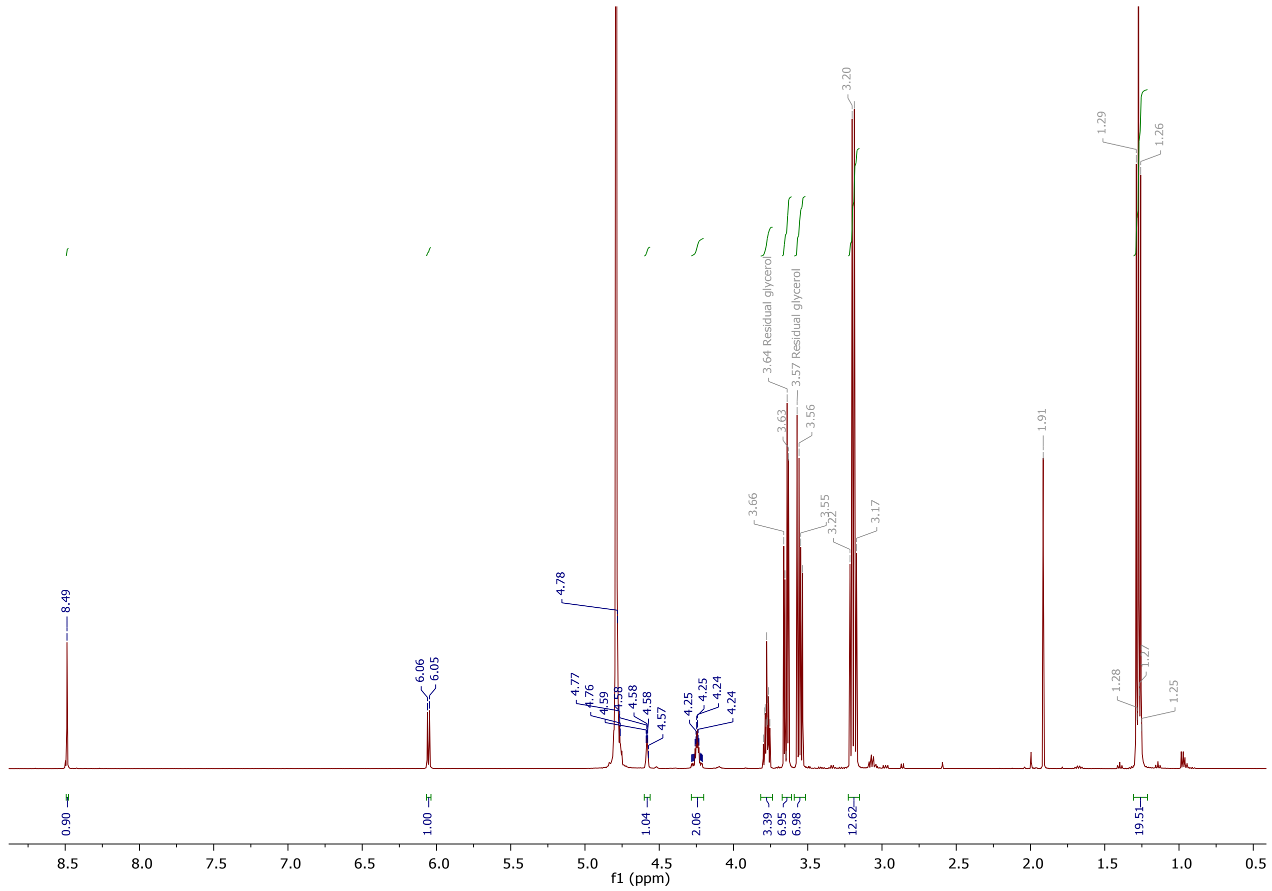

COSY

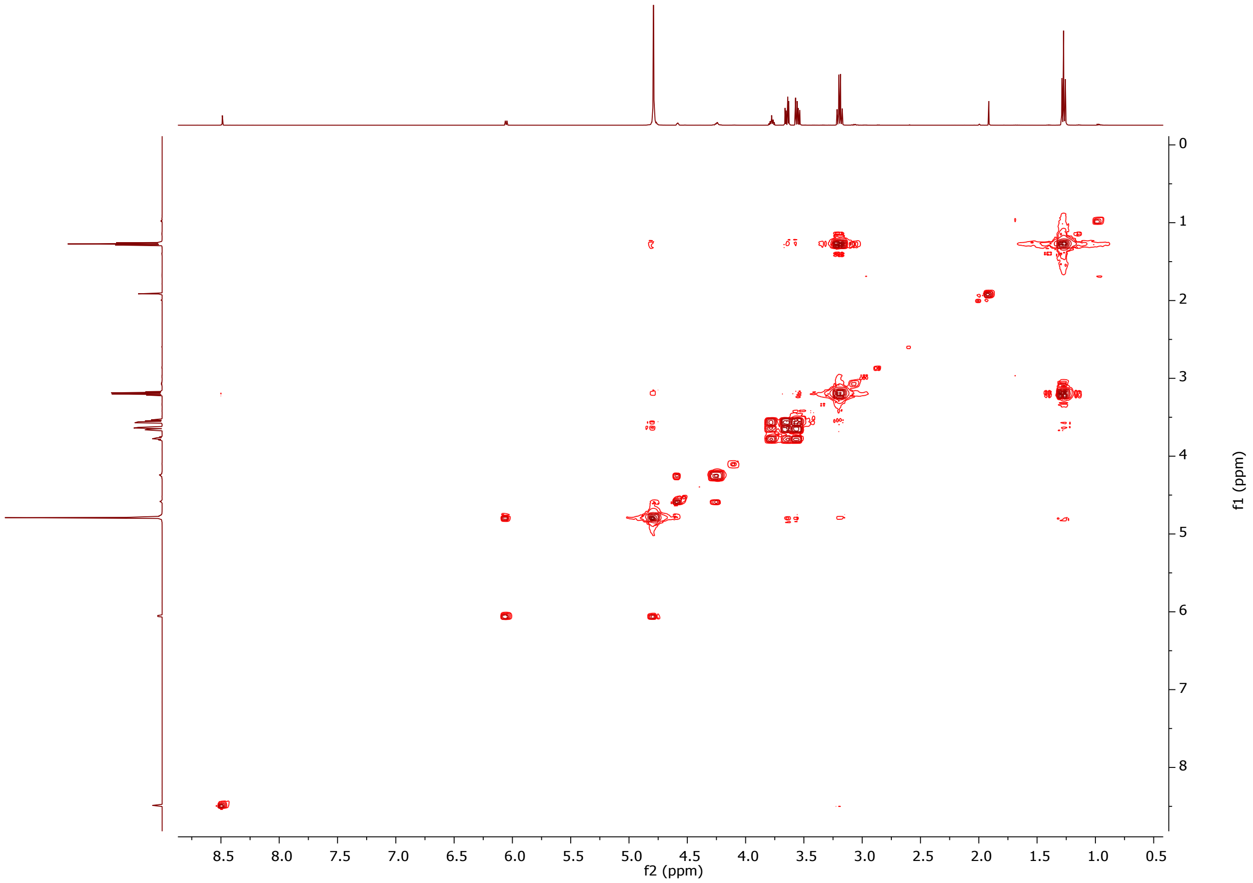

8.5 8.0 7.5 7.0 6.5 6.0 5.5 5.0 4.5 4.0 3.5 3.0 2.5 2.0 1.5 1.0 0.5
f2 (ppm)
0 1 2 3 4 5 6 7 8
f1 (ppm)

^{31}P NMR (202 MHz, D_2O, H_3PO_4, 25^{0}C)

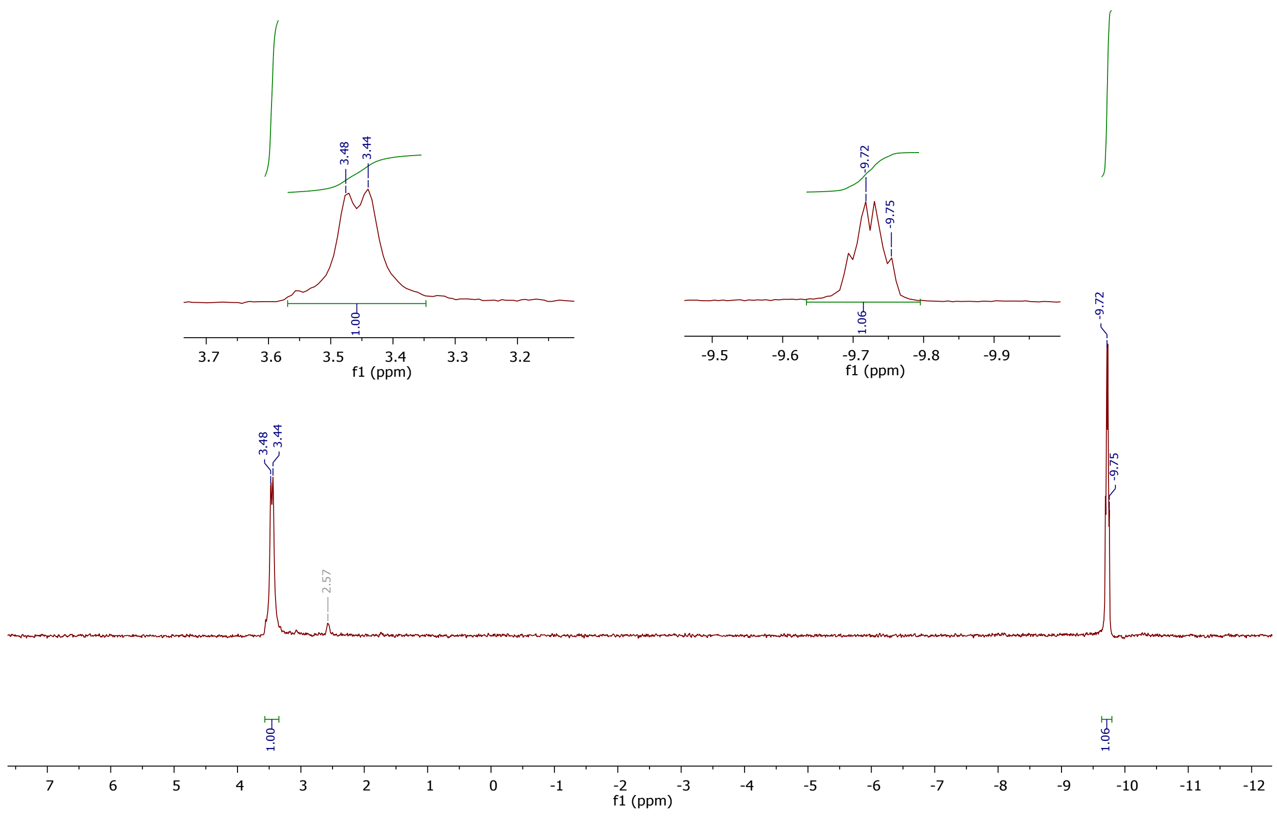

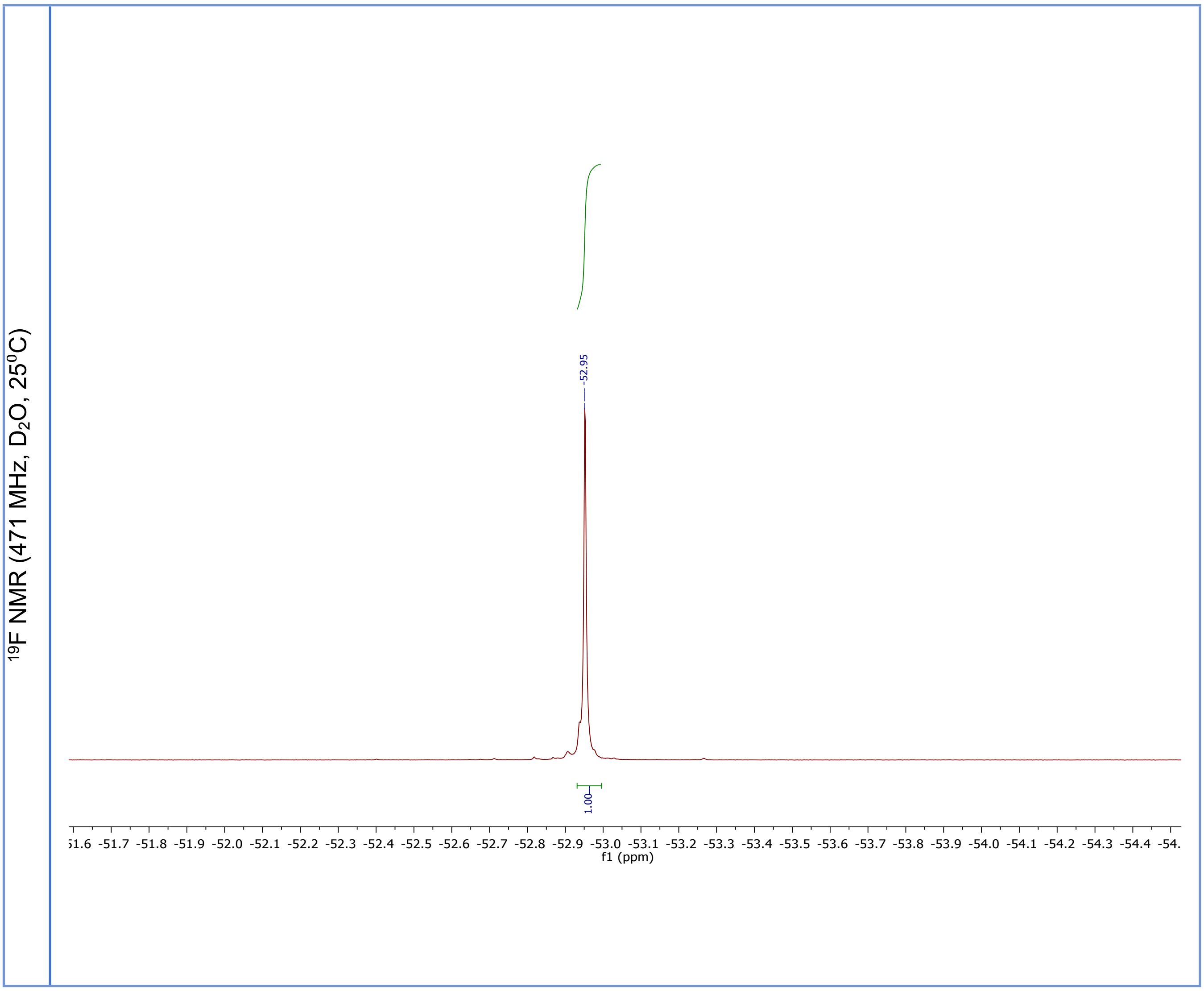

19F NMR (471 MHz, D2O, 25oC)
-52.95
1.00
51.6 -51.7 -51.8 -51.9 -52.0 -52.1 -52.2 -52.3 -52.4 -52.5 -52.6 -52.7 -52.8 -52.9 -53.0 -53.1 -53.2 -53.3 -53.4 -53.5 -53.6 -53.7 -53.8 -53.9 -54.0 -54.1 -54.2 -54.3 -54.4 -54.
f1 (ppm)

Compound 2 : 2-CF_3-PAPS

Chemical structure

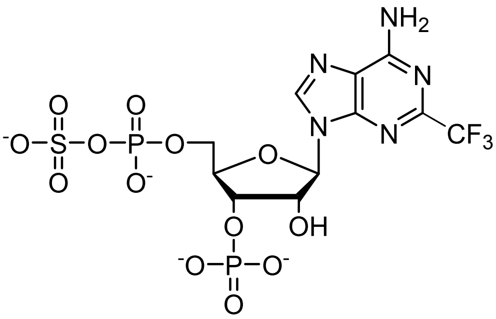

HPLC

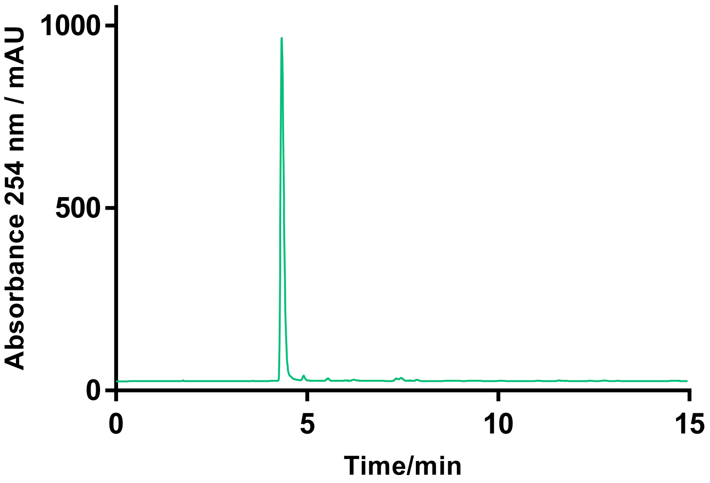

HRMS (-) ESI (Calc. $[M-H]^-$ $C_{11}H_{13}F_3N_5O_{13}P_2S^-$: 573.96634)

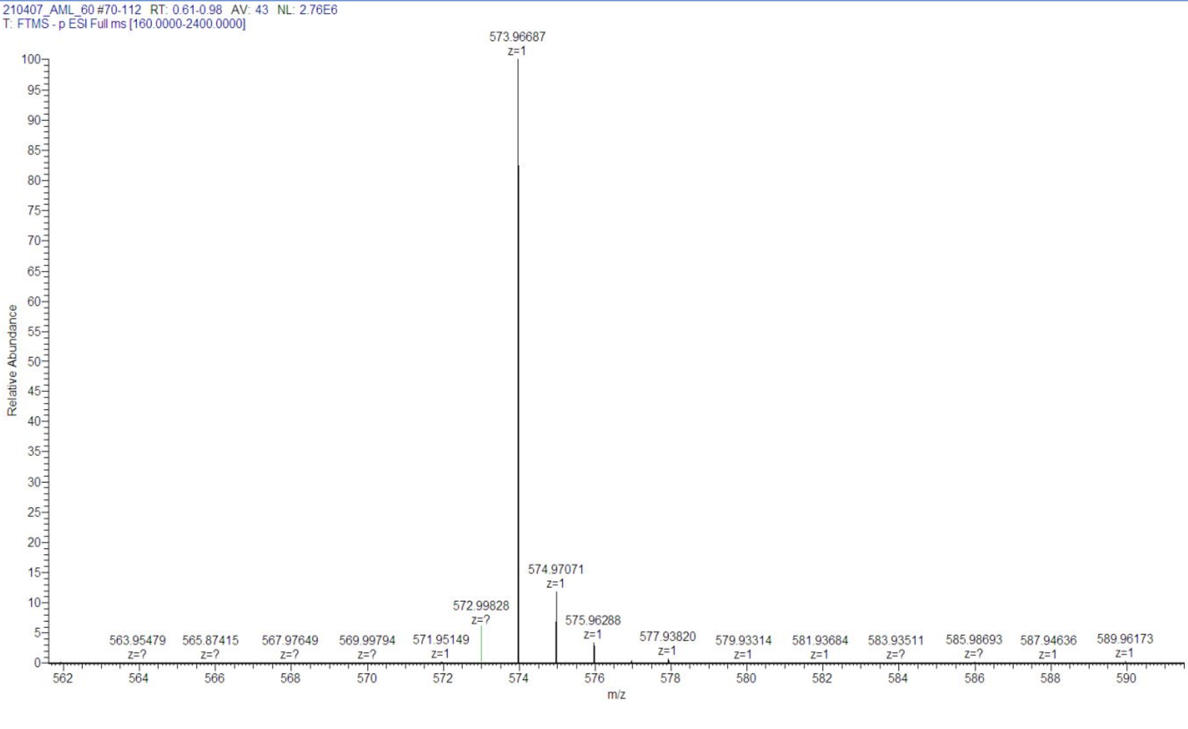

^{1}H NMR (500 MHz, D_2O, 25^0C)

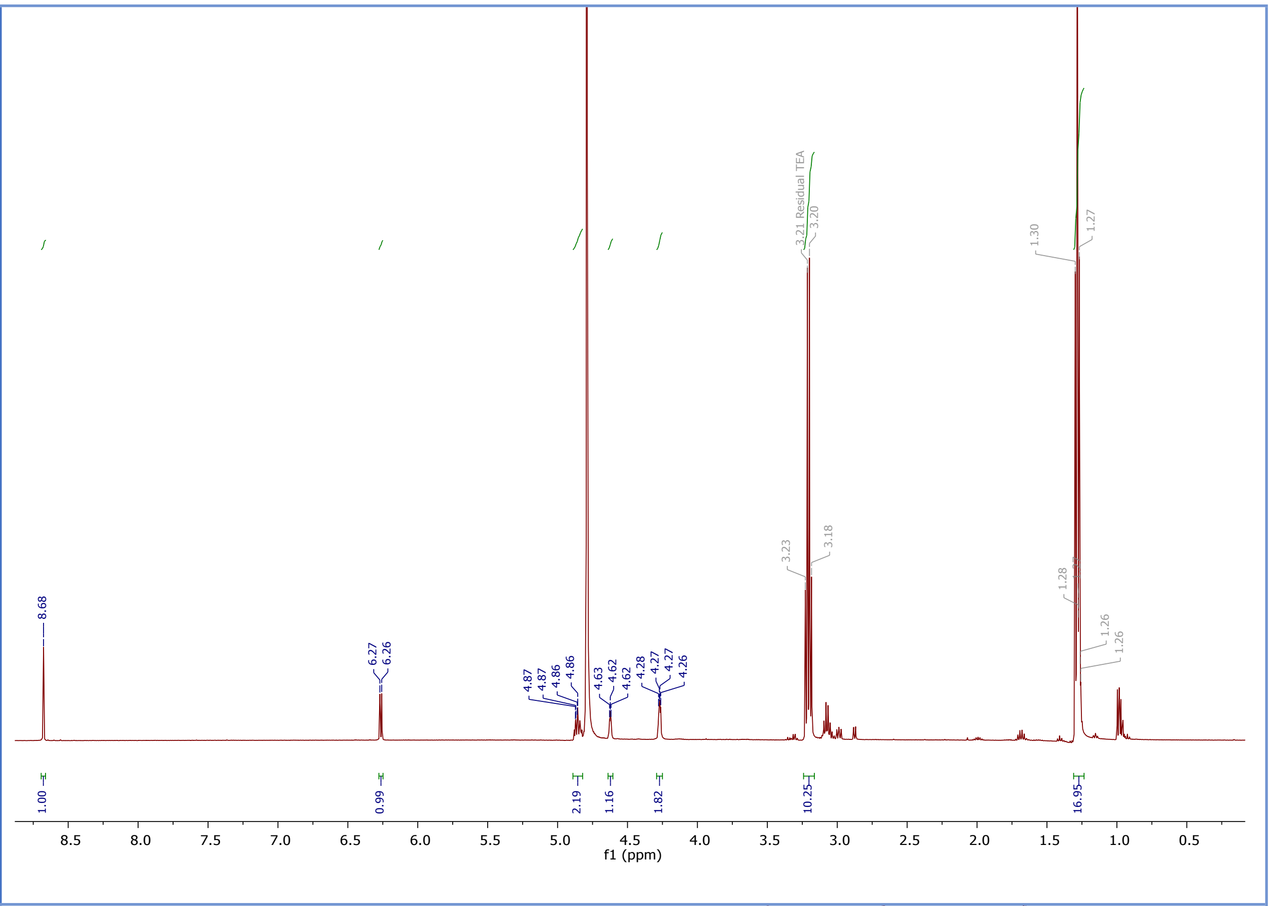

COSY

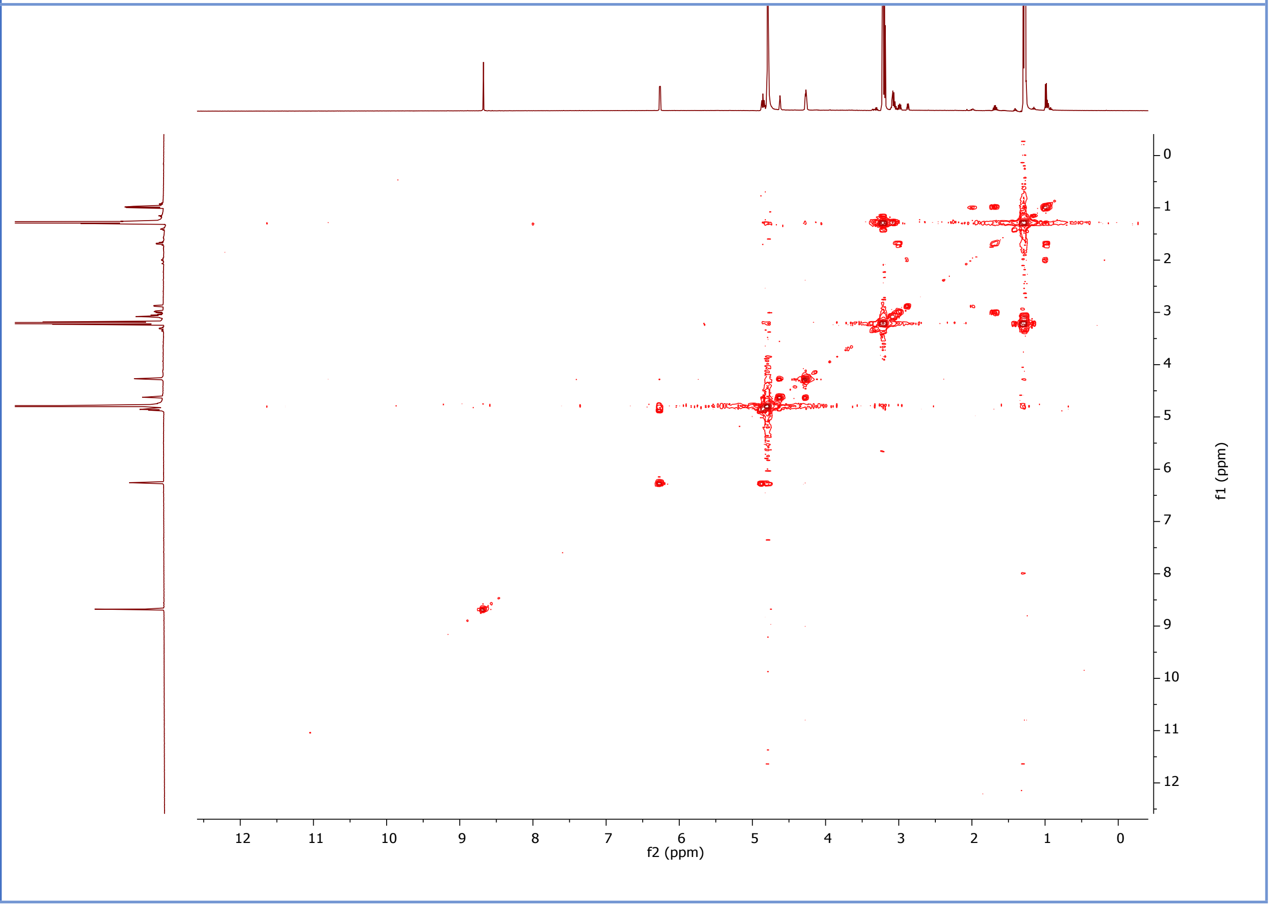

^{31}P NMR (202 MHz, D_2O, H_3PO_4, 25°C)

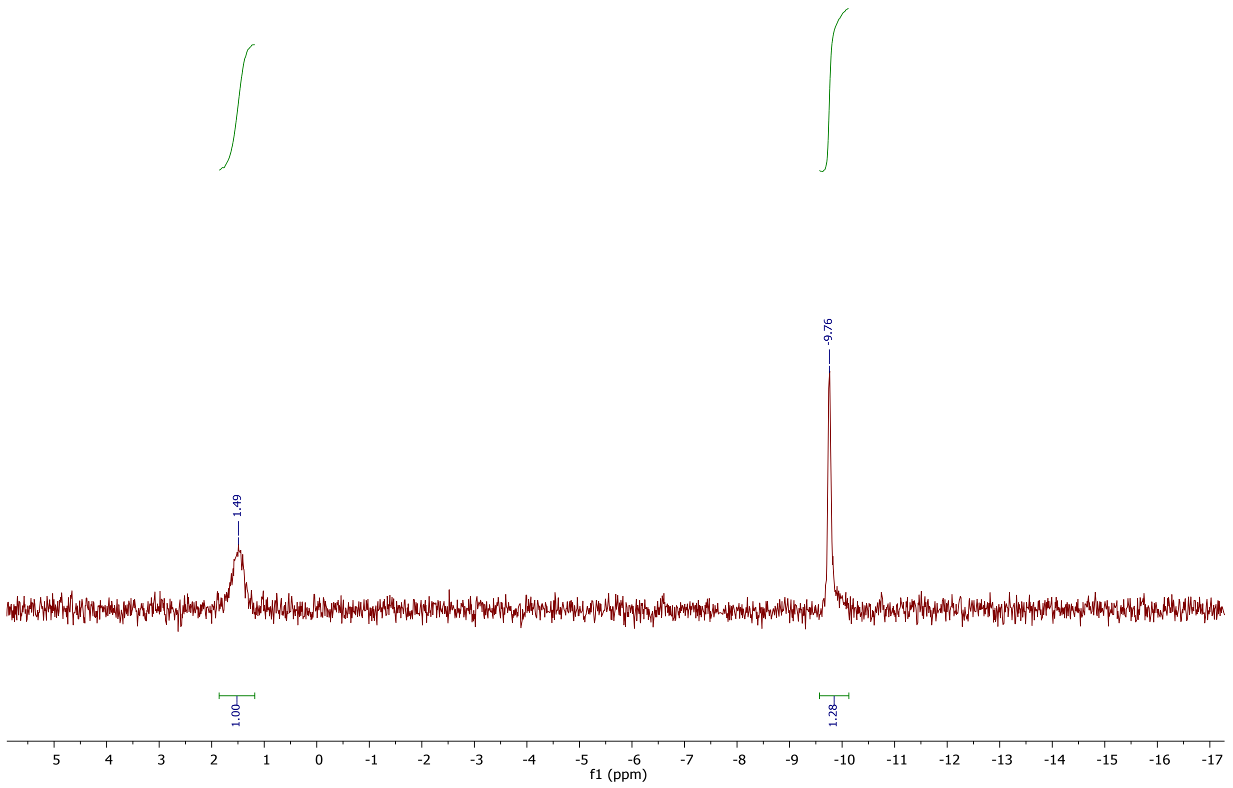

^{19}F NMR (471 MHz, D_2O, 25°C)

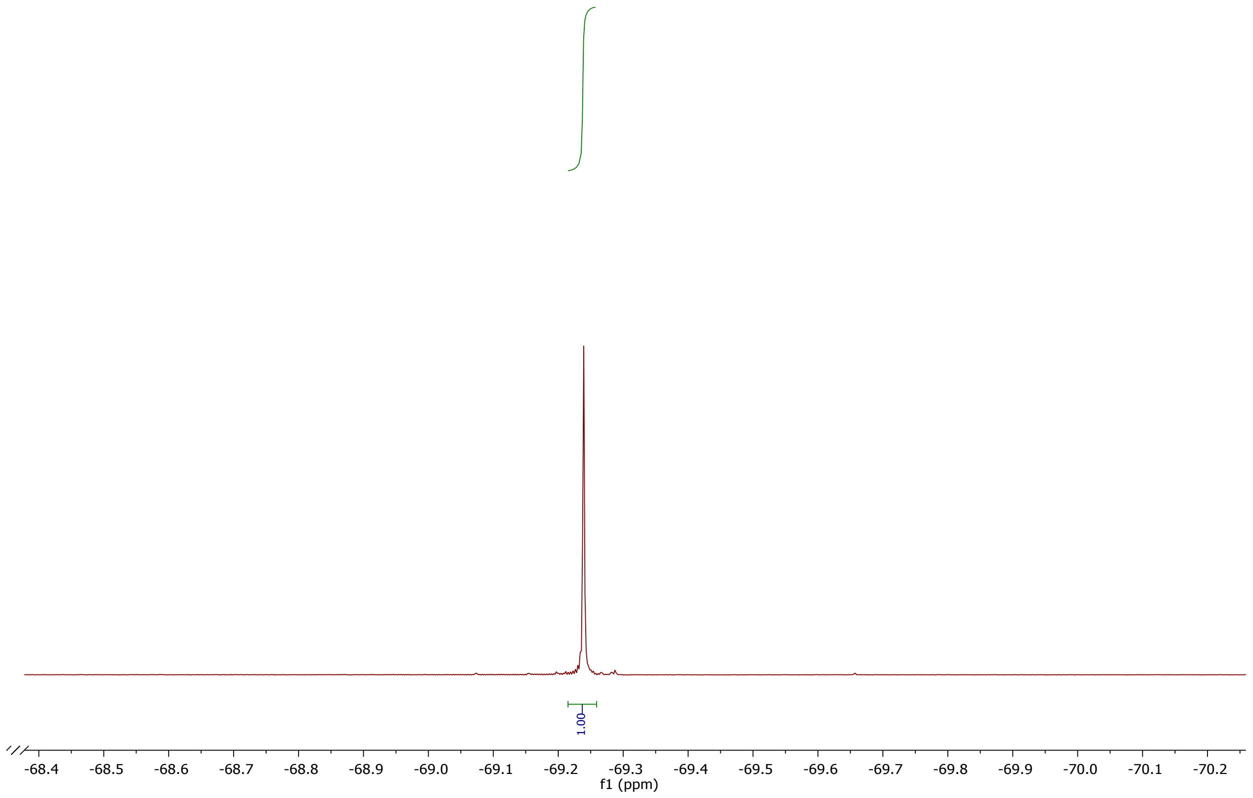

Compound 3 : 8-CF_3-PAPS	
Chemical structure	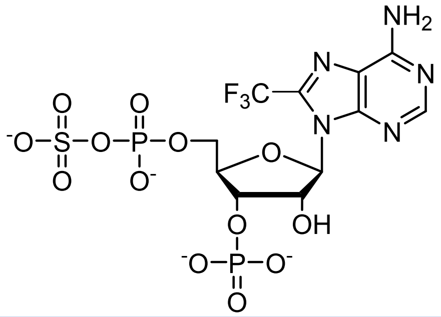
HPLC 254	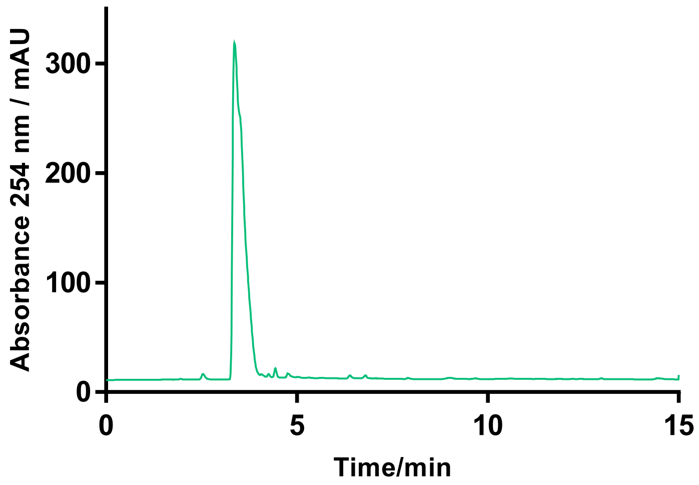
HPLC Ex. 260, Em. 370	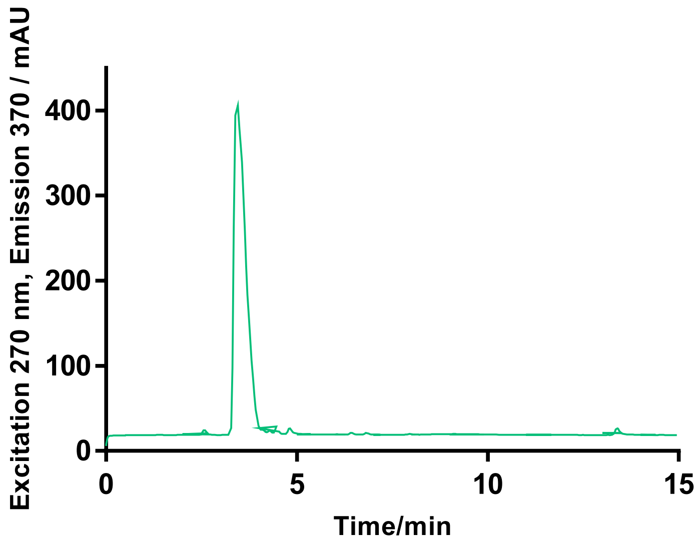

HRMS (-) ESI (Calc. [M-H]$^-$ $C_{11}H_{13}F_3N_5O_{13}P_2S^-$: 573.96634)

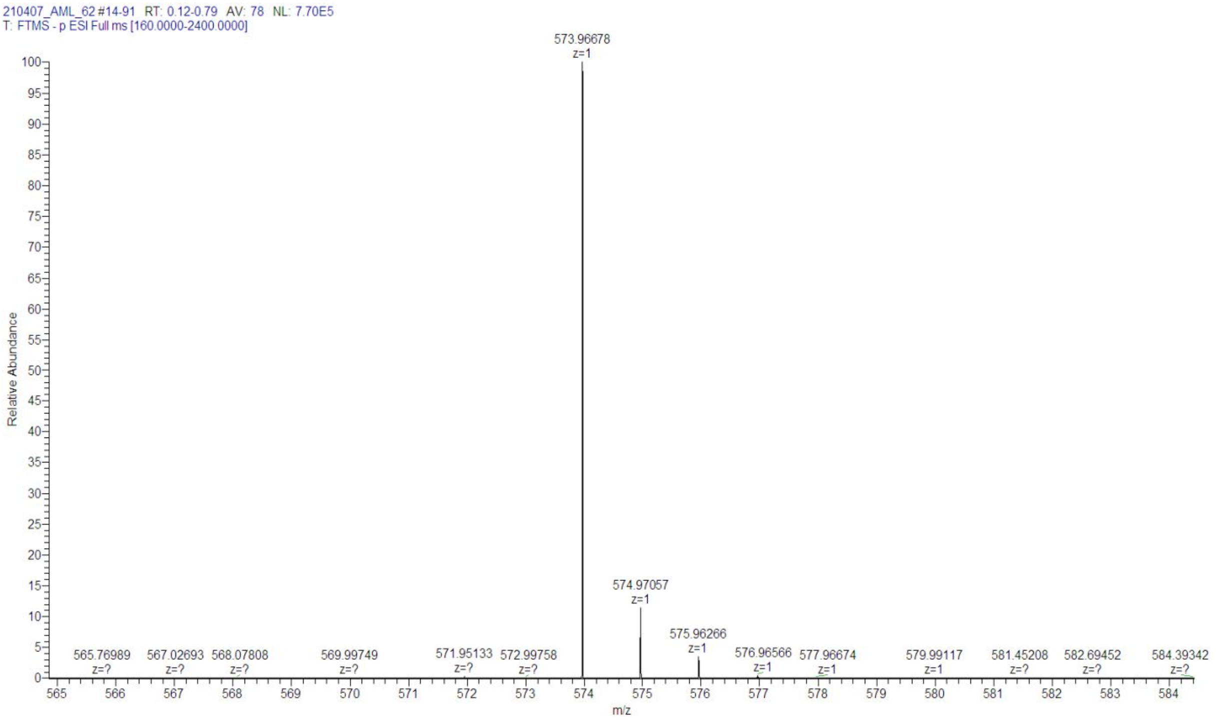

^1H NMR (500 MHz, D_2O, 25^{0}C)

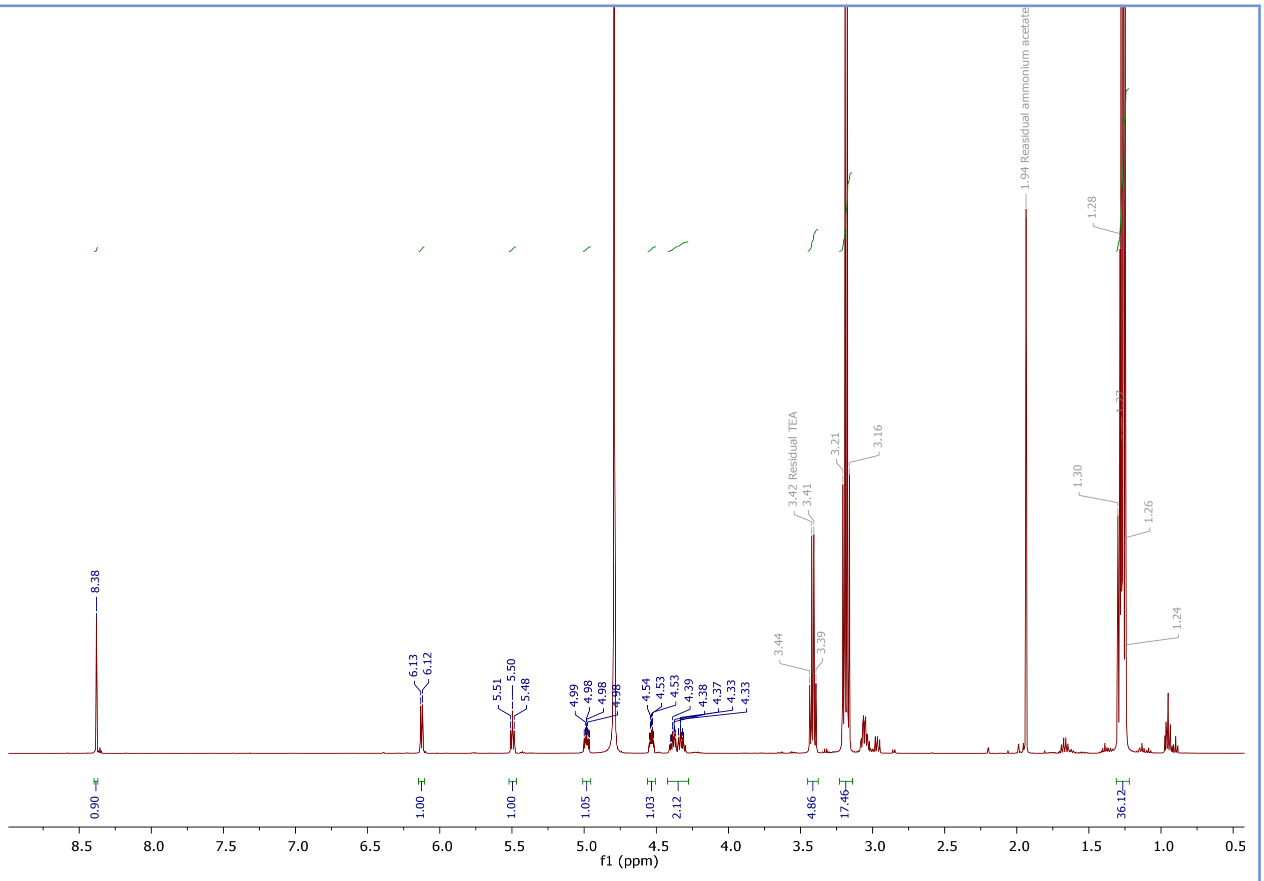

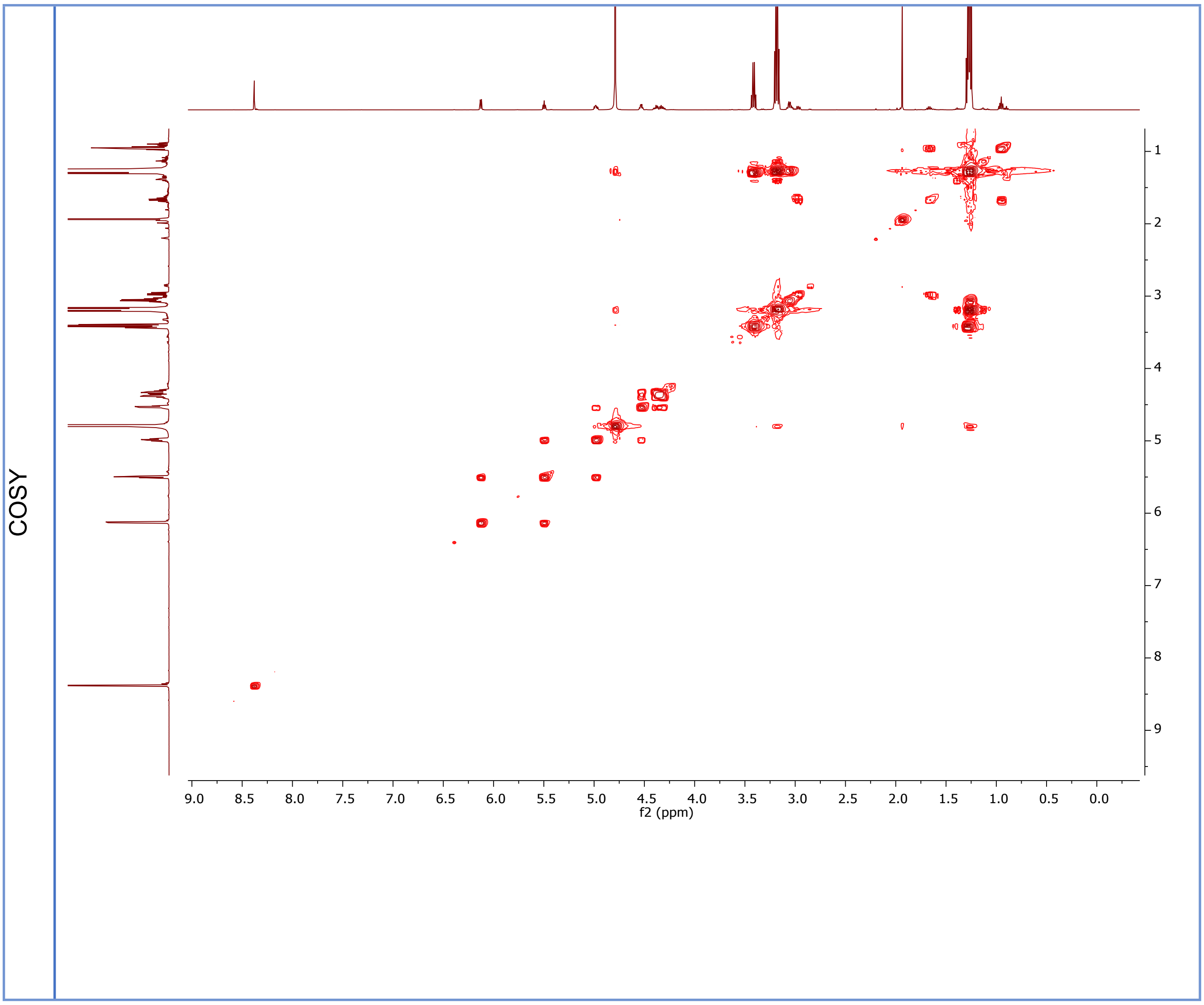
COSY
9.0 8.5 8.0 7.5 7.0 6.5 6.0 5.5 5.0 4.5 4.0 3.5 3.0 2.5 2.0 1.5 1.0 0.5 0.0
f2 (ppm)
1 2 3 4 5 6 7 8 9

^{31}P NMR (202 MHz, D_2O, H_3PO_4, 25^{0}C)

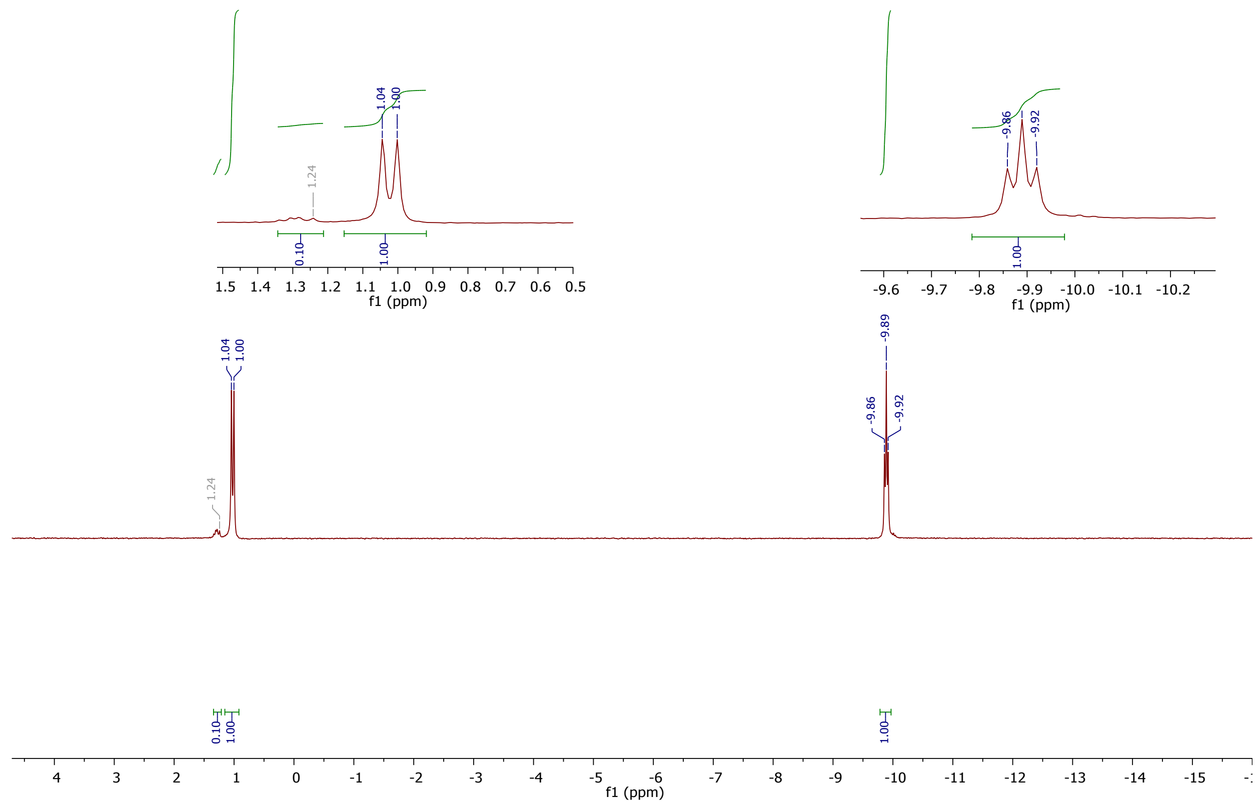

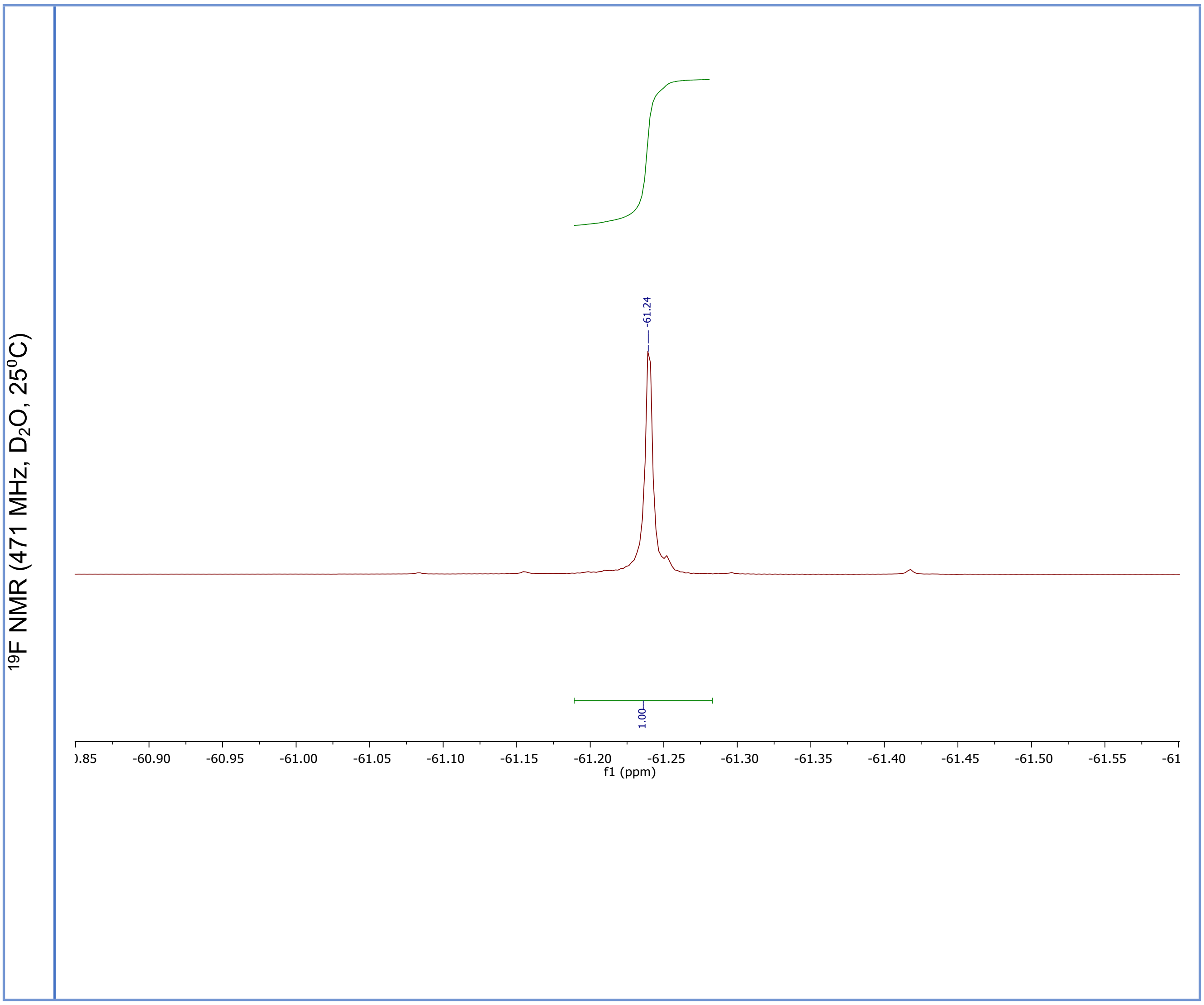

^{19}F NMR (471 MHz, D_2O, 25^{0}C)
-61.24
1.00
f1 (ppm)
